# Professional identity research in the health professions—a scoping review

**DOI:** 10.1007/s10459-022-10171-1

**Published:** 2022-11-09

**Authors:** Marian Cornett, Claire Palermo, Susan Ash

**Affiliations:** Monash Centre for Scholarship in Health Education, Monash University, Faculty of Medicine, Nursing and Health Sciences, Melbourne, Australia

**Keywords:** Health sciences education, Theory of identity, Professional identity, Professional identity theory

## Abstract

Professional identity impacts the workforce at personal, interpersonal and profession levels however there is a lack of reviews of professional identity research across practising health professionals. To summarise professional identity research in the health professions literature and explore how professional identity is described a scoping review was conducted by searching Medline, Psycinfo, Embase, Scopus, CINAHL, and Business Source Complete using “professional identity” and related terms for 32 health professions. Empirical studies of professional identity in post-registration health professionals were examined with health profession, career stage, background to research, theoretical underpinnings and constructs of professional identity being extracted, charted and analysed using content analysis where relevant. From 9941 studies, 160 studies across 17 health professions were identified, with nursing and medicine most common. Twenty studies focussed on professional identity in the five years post-entry to the workforce and 56 studies did not state career stage. The most common background for the research was the impact of political, social and healthcare reforms and advances. Thirty five percent of studies (n = 57) stated the use of a theory or framework of identity, the most common being classified as social theories. Individual constructs of professional identity across the research were categorised into five themes—*The Lived Experience of Professional Identity; The World Around Me; Belonging; Me;* and *Learning and Qualifications*. Descriptions of professional identity are broad, varied, rich and multi-layered however the literature is under theorised with current theories potentially inadequate to capture its complexity and make meaningful contributions to the allied health professions.

## Introduction

Professional identity impacts the workforce at personal, interpersonal and profession levels, yet our understanding of this phenomenon is still developing. In the health professions, literature reviews of professional identity involve single professions, are focussed on students or involve multidisciplinary teams and do not address the range of health professions or their specific issues post registration (Best & Williams, 2019; Jebril, 2008; Maile et al., 2019; Sarraf-Yazdi et al., 2021; Snell et al., 2020; Volpe et al., 2019; Woods et al., 2016; Wyatt et al., 2021a). There has been no synthesis of professional identity research for practising health professionals or attempts to use the different theoretical perspectives described in the literature to deepen our understanding of professional identity and its impact on the health workforce.

The history of identity research is long and complex with many different approaches employed to understand this phenomenon. Identity research can be overwhelming and confusing (Korthagen, 2004)—the term identity has been used by researchers from a range of different fields and a range of paradigms and language, and has been conceptualised from multiple perspectives with connected and overlapping concepts (Brenner et al., 2021; Grootenboer et al., 2006; Kasperiuniene & Zydziunaite, 2019). It is acknowledged that the various subdivisions and categories within the research are somewhat arbitrary, and the different terms and categories used in professional identity research do not refer to orderly, agreed on, and internally consistent sets of ideas, rather their meaning is dependent on the vantage point of the researcher. Despite this, providing a framework to classify identity research is useful to begin to scope the literature in this area. Grootenboer et al. (2006) suggest these approaches fall into three categories based on theoretical underpinnings of the research: (i) individual—psychological/developmental; (ii) sociocultural; and (iii) poststructural perspectives.

*Individual approaches* to identity research originated in Erikson’s psychoanalytic theory of ego identity (Marcia, 1993) and focus primarily on psychological and/or developmental perspectives of the individual. Identity is conceptualised as being located as internal to the individual with identities being regarded as self-determined in response to life experiences (Kroger & Marcia, 2011). For example, in the health professions individual professional identity has been described as an actualisation of one’s morals, values and beliefs (Fagermoen, 1995, 1997; Gomaa, 1999; Peter et al., 2018) giving meaning to one’s self and one’s professional life (Fagermoen, 1995).

*Social approaches* to identity in contrast focus on the interaction of the individual with their social surroundings, both in relationships with individuals (Chen et al., 2011) and groups (Spears, 2011) and with culture in general (Serpe & Stryker, 2011). Tajfel (1979) first conceptualised social identity as being located both internal and external to the individual and developed through social interactions and practices. Social aspects of identity in relation to the group, referred to as collective professional identity, are said to guide professional behaviours and practices through professionalisation (Fagermoen, 1997; Niemi & Paasivaara, 2007). This internalisation of beliefs, values, and behaviours of the profession (Cruess et al., 2015; Jarvis-Selinger et al., 2012) dictates the way in which members of the professional group behave (Abrams & Hogg, 2004; Beddoe, 2011, 2013; Hogg et al., 1995; Smith & Terry, 2003; Terry & Hogg, 1996). Identification with the professional group thus contributes to the development of competent and confident health professionals (Carrillo & Rubel, 2019; Cascón-Pereira et al., 2016; Feen-Calligan, 2012; Findlow, 2012; Ohlen & Segesten, 1998; Sawatsky et al., 2020) influencing the way in which individual professional identity develops and professional practice is enacted (Slay & Smith, 2011).

*Poststructural* approaches challenge the idea of identity being either an individual or social phenomenon (Halualani, 2017; Zembylas, 2003). With the two key concepts of poststructural perspectives being discourse or ‘language in use’ (Cameron & Panovic, 2014) and the subject or self (Foucault, 1982), poststructuralism theorises the self and the social world as socially constructed through discourse (Foucault, 1981). Poststructural approaches to identity interrogate discourse and knowledge fields from which questions of identity are posed and rather than being individual or social posit that identity is located within a broader context embedded in power relations, ideology, and culture and dependent on power and agency (Zembylas, 2003). This broader context includes that of the research as well as the researcher (Harding, 1991). Understanding that whoever defines the problem has a powerful role in shaping the worldview that results from the research (Harding, 1991), interrogating the place from which questions of identity are posed can add to understandings of the social and historical context of practices and discourses in the health professions (Bhabha, 1987). Poststructural approaches attempt to address these underlying assumptions, paradigms and biases that impact the research process at every stage, from concepts and hypotheses selected, to research design, to collection and interpretation of data (Harding, 1991).

Criticism of professional identity research in the health professions has begun to emerge with concerns that critical aspects of professional identity and their associated power relations have not been adequately considered (Tsouroufli et al., 2011; Volpe et al., 2019; Wyatt et al., 2021a; b; Wyatt et al., 2020; Sarraf-Yazdi et al., 2021). Critical approaches to identity compel us to consider not only broader contexts such as discourse and knowledge fields and the positionality of the researcher and the research but to also consider the intersectionality of history, culture, race, socioeconomic status and gender to arrive at a more nuanced and contextual understanding of identity (Halualani, 2017). Consideration and understanding of critical perspectives of professional identity is essential for the well-being, resilience and advancement of health professionals and the health professions. For example, it has been argued that identities based on group membership are utilised for implementing forms of control through a standardised identity (Jemielniak, 2008), for the maintenance of dominant social groups (Tsouroufli et al., 2011; Wyatt et al., 2021a) and for the production and reproduction of dominant ideologies (Apker & Eggly, 2004). This likely contributes to issues with reconciling personal and professional identities, an ongoing task of professionalization (Baldwin et al., 2017; Moorhead et al., 2019; Sharpless et al., 2015; Volpe et al., 2019). For many professionals this is straightforward, however for those whose personal identities or beliefs are at variance with aspects of their professional identity, for example, dominant social groups, dominant professional paradigms or expectations of professional roles, consolidation of professional identity may be problematic (Costello, 2005; Monrouxe, 2010; Volpe et al., 2019; Wyatt et al., 2020, 2021a, 2021b). This can be particularly so as public and social policy as well as public perception shape identity as much as the self-definition of the profession and the professional (Landman & Wootton, 2007). Considering the impact of current challenges in healthcare provision and changes to the way in which health care is delivered (Duckett, 2005; Green et al., 2001; Health Professions Council of Australia, 2005; Sturmberg et al., 2018; Swerissen et al., 2018) understanding individual and collective professional identities has never been more important as inability to reconcile personal and professional identity can contribute to identity dissonance and impact personal and professional health and wellbeing (Costello, 2005; Monrouxe, 2010). To address recent criticism of the professional identity literature and to elucidate different perspectives of professional identity it is important to interrogate why professional identity among health professions has been studied, how, by whom and in whose interest. Synthesising this evidence will provide a more contextual understanding of the body of literature, why it has been studied and in whose interest, which will identify gaps as well as contribute to an appreciation of perspectives that may be impacting our understanding of professional identity and its impact on health workforces. As such we set out to explore the current literature on professional identity across the health professions using a scoping review.

Scoping reviews are effective tools for understanding the extent, distribution and basis of the literature when an area of research is complex or has not been reviewed comprehensively (Arksey & O'Malley, 2005; Mays et al., 2001). Scoping reviews allow for the mapping of theoretical frameworks, concepts and methodologies underpinning an area of research as well as the main sources and types of evidence available (Arksey & O'Malley, 2005; Mays et al., 2001). Categories described by Grootenboer et al. (2006) were used to address questions of theories, frameworks and constructs of profession identity in this review of the health professions literature. Taking into consideration criticisms of inadequate consideration of critical aspects of the research we expanded the category ‘poststructural perspectives’ to ‘poststructural and critical perspectives’, grouping together perspectives which interrogate or disrupt underlying assumptions of the research.

This scoping review is not an effort to produce an accepted standard definition or agreed-upon theoretical perspective for professional identity which may perpetuate power structures. Instead through examining and unpacking *how* professional identity is discussed this scoping review will contribute to our understanding of professional identity across the health professions (Greenhalgh, 2021) and encourage stronger consideration of theoretical perspective, broader contexts and reflexivity in research. Rather than simply mapping the research, this systematic scoping review aims to interrogate the research into professional identity of practising health professionals more fully to include questions of “why” and “in whose interest?” This scoping literature review aims to explore the literature on professional identity, specifically in what disciplines and career stage the evidence is focussed, why the research was undertaken, what theory or framework was used to guide the research and what constructs are used to discuss professional identity. Supporting a more contextual understanding of the body of literature this scoping review will not only identify gaps in the literature and identity perspectives of professional identity that are important across the health professions it will also assist researchers in navigating the complexity of literature across multiple health professions guiding considered, relevant and meaningful approaches to professional identity research.

## Methods

A scoping review, guided by the six-step methodology originally described by Arksey and O’Malley (2005) and expanded by Daudt et al. (2013) and Levac et al. (2010) with reporting guided by PRISMA-ScR (PRISMA extension for Scoping Reviews) (Tricco et al., 2018) was conducted.

### Identifying the research question (step 1)

The scoping review addressed the following question:

How is professional identity described across the health professions literature? Specifically:

- Q 1: Where is most of the literature on professional identity located—by profession and stage of career?
- Q 2: What is the background for research into professional identity in the health professions—why are questions of professional identity being asked?
- Q 3: Which theories of identity form the basis of professional identity research in the health professions literature?
- Q 4. In addition to theories of identity what constructs of identity of professional identity are found in the health professions literature?

### Identifying relevant studies (step 2)

A broad range of sources were searched for literature including multiple electronic databases and hand-searching of reference lists. However, only peer-reviewed empirical studies, including systematic and scoping reviews, and higher degree by research theses were included. Due to the large, rich, complex and heterogenous volume of literature in this field grey literature, i.e., materials and research produced outside of the commercial or academic publishing and distribution channels, was not included in the review. Inclusion and exclusion criteria for the scoping review were developed prior to study selection. Inclusion criteria used in our scoping study related to: type of study; health profession (n = 32); career stage; terminology and focus of the paper. In line with scoping review guidelines (Arksey & O'Malley, 2005; Daudt et al., 2013; Levac et al., 2010) inclusion and exclusion criteria were refined post hoc as we became more familiar with the literature and the various issues which impacted our search (Table 1). All decisions about inclusion/exclusion criteria were reached by consensus between the three authors.

**Table 1:** Final inclusion and exclusion criteria

Inclusion	Exclusion
English	Non-English
Professional identity from the perspective of the health professional;	Professional identity from the perspective of anyone other than the health professional
Empirical research only—all study designs including qualitative, quantitative and mixed methods	Books/chapters; conference proceedings; editorial; commentary; opinion; review; grey literature (materials and research produced by organizations outside of the traditional commercial or academic publishing and distribution channels)
Graduate and post-registration health professions as defined by**:** from the Australian Health Practitioner Regulation Agency (AHPRA) (https://www.ahpra.gov.au/) and *Allied Health* Professions Australia (AHPA) (https://ahpa.com.au/)	Trainee/student/pre-registration; mixed populations—students and health professionals; the public and health professionals
Post hoc clarification—health professions as above, and including: psychologist—educational/school; sex therapists; family therapists; school nurses	Psychologist—organisational and industrial; assistants—allied health, physician’s, dental, paediatric; veterinarians
Post hoc clarification—as long as eligible for health professional registration: doctoral students; academics; clinical educators (practice teachers); healthcare managers	Pharmacy interns; pharmacy technicians; dental hygienist
Post hoc clarification—naturopaths are not registered with AHPRA or listed on AHPA however, included as mainstream as defined by being widely covered by private health insurance in Australia until 2019 (Ooi et al., 2018)	Homeopaths

### Literature search strategy

In conjunction with the Faculty Subject Librarian (see Acknowledgements) search parameters were formulated and Medline; Psycinfo; Embase; Scopus; CINAHL; Business Source Complete were searched on 5 April 2020 by MC. An example of search strategies used is described in “Appendix 1”. Search syntax (e.g., field codes and proximity operators) were modified to suit the individual databases to support answering the research question.

### Study selection (step 3)

Studies from the search of the six databases were downloaded into Endnote (X9) and exported to Covidence (© 2020 Melbourne, Australia) for screening and assessment. Duplicates were removed and remaining titles and abstracts were screened for eligibility against exclusion and inclusion criteria (Table 1). Eligible studies underwent full text screening using the same criteria. All screening was carried out in duplicate by the first author and one of the other authors. Authors were dietitians by profession, with two of the three being experienced researchers and educators in the field and  the third an experienced clinician and researcher. Decisions about inclusion/exclusion for disputed studies were reached by consensus between the three researchers.

Hand searching of reference lists of final included studies did not reveal any further studies.

### Data charting and analysis (step 4)

To comprehensively explore descriptions of professional identity in the health professions literature to address the research questions, key information from the included research studies such as author, year and title data and so on were imported from Covidence to directly pre-populate the charting table. Information was noted and charted in a uniform and systematic way using Microsoft Excel (*Version 1808*, 2019). All studies were sorted and counted with respect to study characteristics.

To answer research question 1 additional information was identified including profession and stage of career of the health professionals. To answer research question 2 the background to the research questions was identified and categorised using conventional content analysis (Hsieh & Shannon, 2005). This involved extraction of statements in each study regarding the purpose, background and/rationale of the research which were assigned preliminary codes by the first author. Using content analysis these codes were grouped into initial categories which were progressively and iteratively grouped into larger categories describing the background to the research or why questions of professional identity being asked.

To answer research question 3 studies in which a theory of identity or professional identity was stated as being used (or in which a novel framework of identity was developed within the study) were identified and theories classified. As described above, research into identity and professional identity originates from a range of traditions and encompasses multiple and varied paradigms and broad classification of theory can help with conceptualisations of these theories and their relationships. Three broad categories of identity theory; *individual, social, and poststructural and critical perspectives,* described discussed in the Introduction were used to classify identity theories from the included studies. To further refine categorisation *Narrative* was included as an additional category as descriptions of narrative-identity in the literature span all three categories (Smith & Sparkes, 2008). A further category, *Environmental*, was devised to accommodate a novel perspective of identity in the health professions literature which emphasizes the influence of the physical environment on identity (Hauge, 2007). Where a study did not explicitly state a theory or develop an identity framework, the authors closely examined the text to in an attempt to infer *categories* of identity or professional identity used in the research.

To ensure the rigour of this part of the coding process, a small number of studies (n = 10) were randomly chosen for comparison coding early in the process by selecting every 15th study of an alphabetical list of studies included in the review. These studies were coded against final categories of identity frameworks by two of the other authors (CP and SA) and resulted in strong agreement between the three coders. In instances where no author could agree on the use of a theory this study was classified as having no overt theory or framework.

To answer question 4, individual *constructs* of professional identity described across all studies were identified and classified using conventional content analysis. (Hsieh & Shannon, 2005) Extracted data were assigned preliminary codes by the first author. These codes or categories were then grouped into initial categories which were progressively and iteratively grouped into larger categories. Categorisation and re-categorisation of the categories within themes as well as linkages between them were further elucidated and developed as coding progressed. To ensure the rigour of this part of the coding process, a small number of studies (n = 8) were randomly chosen for comparison coding early in the process. These studies were free-coded (i.e., did not use the codes already developed) by one of the other authors (CP) and resulted in strong agreement between the two coders. This process was repeated for extracted data on influences on identity. Constructs or themes of identity described within the professional identity literature were charted accordingly.

## Results (step 5)

**Fig. 1:**
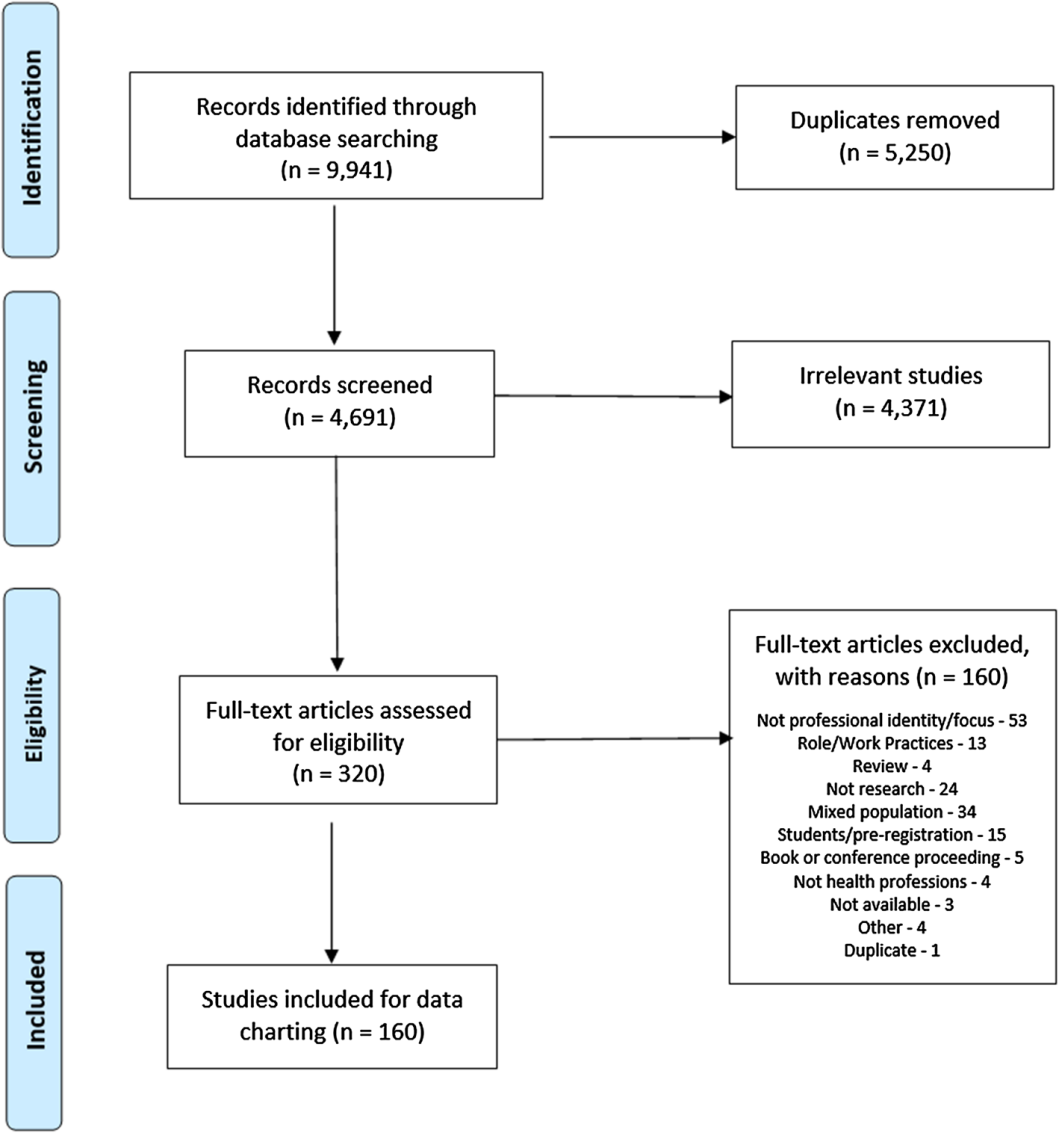
PRISMA diagram depicting search results and selection of included studies

The search yielded 9,941 articles and after duplicates were removed 4,691 articles were screened by title and abstract for eligibility against the selection criteria. Three hundred and twenty papers were deemed appropriate for full text-screening with a final 160 studies deemed eligible for inclusion in this scoping review (Fig. 1). For a complete list of the 160 references included in the review see “Appendix 2”.

### Q 1: where is most of the literature on professional identity located—by profession and stage of career?

The largest number of studies were from the United States (48), United Kingdom (26) and Australia (21) with the majority of studies focusing on the disciplines of nursing (59) and medicine (38) (Table 2). The remaining 63 studies included other specialities.

**Table 2:** Study characteristics

		Studies (no. of 160)
Country	US	48
	UK	26
	Australia	21
	New Zealand	7
	Canada	9
	Japan	5
	Netherlands	4
	Sweden	4
	Norway	3
	Spain	3
	Greece	3
	Finland	3
	Turkey	3
	Italy	3
	China	2
	Israel	2
	Belgium; Brazil; Czech Republic; Denmark; Ireland; Iran; Hong Kong; Malaysia; Portugal; Singapore; Sudan: Switzerland; UK & Australia; multiple countries	1 each, total 14
Publication type	Journal article	135
	Thesis	25
Health profession	Nurse	59
	Doctor	38
	Social worker	18
	Counsellor	9
	Occupational therapist	8
	Psychologist	6
	Pharmacist	4
	Allied health professional	2
	Art therapist	2
	Dentist	2
	Doctor and nurse	2
	Psychoanalyst	2
	Complementary & alternative medicine (Chinese medicine, chiropractic, osteopathy); occupational therapy and physiotherapy clinician educators; optometrist; osteopath; physiotherapist; psychiatrist & psychologist; radiographer; youth worker	1 each, total 8
Stage of career	< 1 year	7
	1–5 years	20
	6–10 years	14
	10–20 years	5
	> 20 years	2
	Various	55
	Not stated	56
	N/A (Text analysis)	1

Fifty-five studies included health professionals at various stages of their careers. Twenty of the 104 studies where career stage was stated included health professionals within 1–5 years of registration and 14 studies looked at professionals 6–10 years post-registration.

### Q 2: what is the background to the research into professional identity in the health professions?

Analysis of the research identified nine categories rationalising the research involving professional identity and related to political, social and healthcare reforms and advances; support of professional identity development and maintenance; understanding professional identity; understanding boundary crossing; for education and learning; support of recruitment and retention; exploration of dominant paradigms; enhancing organisational engagement; and improving patient care (Table 3). Forty-five studies (28%) were concerned with the political, social and healthcare reforms and advances. This included the *impact on* professional identity (35 or 22%) (Bertin & Pantalone, 2019; Blomberg, 2016; Bludau, 2017; Bochatay, 2018; Brunton, 2017; Carpenter & Platt, 1997; Cascón-Pereira et al., 2016; Currie et al., 2010; Dahl & Clancy, 2015; de Meis et al., 2007; Feen-Calligan, 2012; Frechette et al., 2020; Furtaw, 2004; Gent, 2017; Gomaa, 1999; Handy et al., 2020; Hendrikx, 2018; Hurley, 2009; Iglesias & De Bengoa Vallejo, 2011; Kyratsis et al., 2017; Larsson et al., 2009; McMurray & Pullen, 2008; Mishra et al., 2012; O’Shea & McGrath, 2019; Ocek & Vatansever, 2014; Piil et al., 2012; Porter & Wilton, 2019; Sanders, 2019; Snelgrove, 2009; Takashima & Saeki, 2019; Thompson, 2005; Thomson et al., 2014; Vincifori & Molinar, 2014; Wiles & Vicary, 2019; Zufferey, 2012), the *role of* professional identity in adjusting to these reforms or advances (8) (Bertrand, 2009, 2010; Dadich et al., 2015; Deppoliti, 2003; Franco & Tavares, 2013; Gregg & Magilvy, 2001; Hammond et al., 2016; Wright, 2007) as well as the broader impact of political and social reform on recruitment and retention of health care workers through professional identity effects (2) (Allen, 2011; Deppoliti, 2008).

**Table 3:** Categories describing the background to professional identity research in the health professions

Categories	Number of studies of 160 (%)
Political, social and healthcare reforms and advances	45 (28)
Supporting professional identity development and maintenance	37 (23)
Understanding professional identity	25 (16)
Boundary crossing	19 (12)
Education and learning	12 (8)
Recruitment and retention	11 (7)
Dominant paradigms	9 (6)
Organisational engagement	1 (1)
Improving patient care	1 (1)

Supporting professional identity development and maintenance was the background to research involving professional identity in 37 studies (23%). Sub-themes included supporting the development and maintenance of professional identity generally (5) (Bartlett, 2008; Branch & Frankel, 2016; Fleit, 2008; Forenza & Eckert, 2018; Harris & Guillemin, 2015), supporting the development and maintenance of professional identity of the profession or speciality (15), (Healey, 2010; Hurley & Lakeman, 2011; Iwasaki et al., 2018; Karpetis, 2014; Lafleur, 2007; Mackay & Zufferey, 2015; Mellin et al., 2011; Moorhead et al., 2016; Mousazadeh et al., 2019; Salim & Elgizoli, 2016; Swickert, 1997; Tahim, 2015; Thompson et al., 2018; Zhang et al., 2015) with four specifically in respect to professions regarded as having a poor reputation or low recognition (Leigh, 2014; Mallon, 2018; Morriss, 2014; Sercu et al., 2015). Supporting professional identity development and maintenance in relation to boundary crossing was also a reason for research involving professional identity. Boundary crossing included into new roles or sub-specialities (9) (Barraclough, 2014; Carra et al., 2017; Carrillo & Rubel, 2019; Chan et al., 2018; Croft et al., 2015a, 2015b; Hazen et al., 2018; Hedenskog et al., 2017; Hercelinskyj et al., 2014) and into academia (4) (Ennals et al., 2016; Findlow, 2012; Smith & Boyd, 2012; Stone et al., 2002). Twenty-five studies (16%) were concerned with understanding the experience or perception of professional identity (9) (Elvey et al., 2013; Fagermoen, 1997; Kantek & Şimşek, 2017; Kluijtmans et al., 2017; MacIntosh, 2002, 2003; Ngai, 2007; Niemi & Paasivaara, 2007; Peter et al., 2018), its construction or influences on its development (9) (Chow et al., 2018; Dombeck, 2003; Estrella, 2010; Fagermoen, 1995; Fitzgerald & Teal, 2004; Hinojosa, 2012; Hinojosa & Carney, 2016; Kumpusalo et al., 1994; Real et al., 2009), the role of the organisational identity in professional identity (4) (Barbour & Lammers, 2015; Chang, 2012; Curtis & Day, 2013; Salvatore et al., 2018); and the role of emotion (1) (Cascón-Pereira & Hallier, 2012). Boundary crossing, both the *impact on* and *role of* professional identity was the reason behind of 19 or 12% of studies being conducted (Berghout et al., 2020; Brosnan & Cribb, 2019; Devery et al., 2018; Divall, 2015; Ferrell, 2017; Koskiniemi et al., 2019; Kunhunny & Salmon, 2017; Martin et al., 2020; McKenzie & Williamson, 2016; McNamara, 2010; Meyer et al., 2015; Ng et al., 2018; Ogilvie, 2012; Ong et al., 2019; Owens, 2018; Pape et al., 2018; Pottie et al., 2009; Pratt et al., 2006; Reyes Villagomeza, 2019) including both boundary crossing to academia and clinical teaching and hybrid roles, particularly clinical-management roles. The impact of education and learning, qualifications and credentials on professional identity and professional capital was investigated in 12 or 8% of studies (Arai et al., 2017; Beckett & Gough, 2004; Beddoe, 2013, 2015; Birks et al., 2010; Blouin, 2018; Clandinin & Cave, 2008; Foster & Roberts, 2016; Hansen et al., 2019; Sawatsky et al., 2018; Sawatsky et al., 2020; Sims, 2011) and the role of professional identity on recruitment and retention of workforce (9) (Becker, 2013; Cowin et al., 2008; Jiang et al., 2019; Karanikola et al., 2018; Landis et al., 2020; Lévesque et al., 2019; McCrae et al., 2014; Moorhead, 2019; Sabanciogullari & Dogan, 2017), including through its role in managing work stress and burnout (2) (Diede, 2018; Fragkiadaki et al., 2019). The impact of dominant paradigms such as the biomedical paradigm of health or dominant paradigms of practice, e.g., cognitive behavioural therapy, were also given as context for research involving professional identity. Again, both the *impact on* (7) (Hanson, 2009; Ka-Hi et al., 2019; Lotan, 2019; Motoike, 2003; Nicacio et al., 2016; Schubert et al., 2020; Yagil & Medler-Liraz, 2015) as well as *the role of* professional identity (2) (Apker & Eggly, 2004; Bentley et al., 2018) was of interest in studies. Organisational engagement of health professionals (Baathe & Norbäck, 2013) and improvement of patient care (Barone & Lazzaro-Salazar, 2015) was the background to one study each.

### Q 3: which theories of identity form the basis of professional identity research in the health professions literature?

The majority of studies (131 or 82%) utilised qualitative methods, with the remaining using quantitative (20 or 12%) and mixed methods (9 or 6%) (Table 4). A wide variety of theoretical and methodological approaches were applied (Table 4). Constructionism and phenomenology were used in 25 and 16 studies respectively and 13 studies took a grounded theory approach. The remaining studies using symbolic interactionism (6), interpretivism (5), criticalism (4), constructionism with a critical perspective (3); and poststructuralism (2). One study each used feminism; standpoint feminism; poststructuralism/feminism; poststructuralism (Bourdieu); social realism; existential phenomenology; or multiple theories (Bourdieu/feminism/cultural theory). Seventy-nine studies (49%) made no comment on overall theoretical framework.

**Table 4:** Theoretical perspectives of studies

		Number of studies of 160
Theoretical approach of overall study	Qualitative	131
	Quantitative	20
	Mixed method	9
Stated theoretical frameworks of overall study	Constructionism	25
	Phenomenology	16
	Grounded theory	13
	Symbolic interactionism	6
	Interpretivism	5
	Criticalism	4
	Constructionism (critical perspective)	3
	Poststructuralism	2
	Feminism; standpoint feminism; poststructuralism/feminism; poststructuralism (Bourdieu); social realism; existential phenomenology; multiple theories (bourdieu/feminism/cultural theory)	I each, total 7
	Not stated	79
Stated theories & frameworks of identity by category	Social	29
	Poststructural and critical	10
	Narrative	**7**
	Individual	4
	Environmental	1
	Combination of theories	4
	Developed theories of professional identity	2
Inferred categories of theories & frameworks	Social	73
	Narrative	10
	Poststructural and critical	6
	Individual	3
Not able to be inferred		11

### Stated theories and frameworks of identity by category

Stated theories and frameworks were identified in the following categories—*individual*, *social*, and *poststructural and critical*. *Combinations of theories* and *theories developed* through the research were also categorised. As described above, the categories *narrative,* spanning all three main categories (Smith & Sparkes, 2008), and *environmental,* accommodating a novel perspective of identity (Hauge, 2007), were included.

Twenty-nine studies utilised theories or frameworks which were categorised under *Social*. Theories and frameworks included Social Identity Approaches including Social Identity Theory and Self-Categorisation Theory (9) (Blouin, 2018; Cascón-Pereira & Hallier, 2012; Cascón-Pereira et al., 2016; Chang, 2012; Jiang et al., 2019; Ka-Hi et al., 2019; Mallon, 2018; Ogilvie, 2012; Salvatore, 2018), self-concept (1) (Cowin et al., 2008) and professional self-concept (2) (Kantek & Şimşek, 2017; Karanikola et al., 2018). Two further studies had a situated learning perspective with identity framed within Wenger’s Community of Practice framework (2) (Forenza & Eckert, 2018; Smith & Boyd, 2012). One study each employed Mead’s Symbolic Interactionism (Fagermoen, 1995), Social Cognitive Theory (Sawatsky et al., 2020), Dubar’s Professional Identities (Nicacio et al., 2016), Relational and Social identity and Identification (Currie et al., 2010), Professional Self-Description (Hedenskog et al., 2017), The Occupational Perspective of Health framework (OPH) (Ennals et al., 2016) and Embedded Intergroup Relations Theory (Furtaw, 2004). Social approaches related to role included Identity Theory (5) (Allen, 2011; Hercelinskyj et al., 2014; Matsui et al., 2019; Ong et al., 2019; Sercu et al., 2015), Role-exit Theory (2) (Bertrand, 2009, 2010) and Positioning Theory (1) (Snelgrove, 2009). Ten studies were categorised as *Poststructural and Critical* and included discursive construction of professional identity (4) (Apker & Eggly, 2004; Mackay & Zufferey, 2015; Real et al., 2009; Schubert et al., 2020), Bourdieu’s Habitus (1) (O’Shea & McGrath, 2019), Anzaldúa's (1987) Borderlands Theory (2) (Hinojosa, 2012; Hinojosa & Carney, 2016), Performance Studies (1) (Chow et al., 2018), Sociopolitical Professional Identity (1) (Motoike, 2003), Robert’s (2000) Model of Identity Development and Oppressed Group Behaviour (1) (Birks et al., 2010). Seven studies were classified under the *Narrative* heading and included Narrative Identity—dialogic (3) (Barone & Lazzaro-Salazar, 2015; Kluijtmans et al., 2017; Morriss, 2014), Narrative identity theory (1) (Divall, 2015), Narrative identity (moral Identity) (1) (Peter et al., 2018) and narrative identity—‘thick social relational focus’ or performative perspective (Barraclough, 2014) and generally a narrative approach (not defined) (Berghout et al., 2020). Four studies used theories or frameworks categorised under *Individual* including a developmental perspective (1) (Fitzpatrick, 2004), Possible Selves (1) (Bartlett, 2008) and one each encompassing learning frameworks of identity related to the individual—Mezirow’s Phases of Transformative Learning (Sawatsky et al., 2018) and Illeris’ Transformative Learning and Identity theory (Owens, 2018). Place-Identity Theory (Harris & Guillemin, 2015) was categorised under *Environmental* with one study in this category. Four studies indicated the use of a *combination of theories*—Identity Theory and Social Identity Theory (1) (Mishra et al., 2012), Social Identity Theory and Bourdieu perspectives (1) (Beddoe, 2013), Heidegger's Principle of Identity (being is always the being of a being) and a developmental framework (1) (Ferrell, 2017), and Career Theory and a developmental framework (1) (Gomaa, 1999). Two further studies were identified which comprised *theories or perspectives which were developed* as part of the study—Worker in Environment (Carpenter & Platt, 1997) and a social theory of professional identity formation (Becker, 2013).

### Inferred categories of theories and frameworks of identity

One hundred and three studies did not specifically state theories or frameworks of identity used in the research. Four broad categories of identity were inferred from a secondary analysis—*individual*, *social*, *narrative*, and *poststructural and critical and perspectives*. Seventy-three studies of 103 studies were classified as *Social* in three subcategories. Thirty studies were identified as being focussed on group membership in their investigation (Barbour & Lammers, 2015; Bochatay, 2018; Brosnan & Cribb, 2019; Carrillo & Rubel, 2019; Croft et al., 2015a, 2015b; Curtis & Day, 2013; Elvey et al., 2013; Feen-Calligan, 2012; Findlow, 2012; Franco & Tavares, 2013; Gregg & Magilvy, 2001; Hammond et al., 2016; Koskiniemi et al., 2019; Kyratsis et al., 2017; Lafleur, 2007; Lévesque et al., 2019; McCrae et al., 2014; Mellin et al., 2011; Meyer et al., 2015; Moorhead et al., 2016; Moorhead, 2019; Mousazadeh et al., 2019; Niemi & Paasivaara, 2007; Porter & Wilton, 2019; Reyes Villagomeza, 2019; Sabanciogullari & Dogan, 2017; Stone et al., 2002; Tahim, 2015; Thomson et al., 2014) whilst 28 studies focussed on role (Baathe & Norbäck, 2013; Dadich et al., 2015; Diede, 2018; Fleit, 2008; Frechette et al., 2020; Handy et al., 2020; Hansen et al., 2019; Hanson, 2009; Hazen et al., 2018; Hendrikx, 2018; Iglesias & De Bengoa Vallejo, 2011; Kumpusalo et al., 1994; Kunhunny & Salmon, 2017; Landis et al., 2020; Martin et al., 2020; McKenzie & Williamson, 2016; Pape et al., 2018; Piil et al., 2012; Pottie et al., 2009; Pratt et al., 2006; Salim & Elgizoli, 2016; Sanders, 2019; Sims, 2011; Swickert, 1997; Thompson et al., 2018; Wright, 2007; Yagil & Medler-Liraz, 2015; Zhang et al., 2015). A further 14 studies were not focussed on either group membership or role and classified as *Social—General* (Beckett & Gough, 2004; Beddoe, 2015; Bentley et al., 2018; Bertin & Pantalone, 2019; Foster & Roberts, 2016; Hurley, 2009; Hurley & Lakeman, 2011; Iwasaki et al., 2018; Larsson et al., 2009; Lewis, 2004; Lotan, 2019; MacIntosh, 2002, 2003; Takashima & Saeki, 2019; Zufferey, 2012). This included one study which in which societal expectations around gender was the focus of professional identity (Lewis, 2004) and one study in which situated learning was identified as the focus (Beckett & Gough, 2004). Narrative perspectives of professional identity were identified in 10 studies (Blomberg, 2016; Brunton, 2017; Carra et al., 2017; Clandinin & Cave, 2008; Dahl & Clancy, 2015; de Meis et al., 2007; Dombeck, 2003; Fragkiadaki et al., 2019; Karpetis, 2014; Leigh, 2014) and poststructural and critical perspectives in 6 studies (Bludau, 2017; Gent, 2017; McMurray & Pullen, 2008; McNamara, 2010; Ngai, 2007; O’Shea & McGrath, 2019). Individual perspectives were identified in three studies including psychological/developmental approaches theories (Branch, 2016; Chan et al., 2018) and learning impacting the individual (1) (Arai et al., 2017).

Categories of identity frameworks were not able to be inferred and assigned in 11 (7%) of the studies.

### Q4. In addition to theories of identity what constructs of professional identity are found in the health professions literature?

Five major themes containing 37 categories of constructs of professional identify were determined from the health professions literature (Table 5). Note that studies may contain multiple constructs of professional identity. Constructs of professional identity linked to references in the health professions literature are presented in “Appendix 3”.

**Table 5:** Constructs of professional identity in the health professions literature

		No. studies of 160 (%)
The lived experience of PI	Becoming from performing	83 (52)
	Knowing from practicing	56 (35)
	Witnessing experiences of others through relationships	30 (19)
	Personal experiences impacting interactions with clients	7 (4)
	Practising	23 (14)
	Philosophy of practice	38 (24)
	Visibility of practice	14 99)
	Autonomy in practice	29 (18)
	Role	86 (54)
The world around me	Workplace and the organisation	62 (39)
	Political, social and healthcare reforms and advances	21 (13)
	Professional hierarchies	44 (28)
	Dominant paradigms	44 (28)
	Knowledge claims	22 (14)
	Health professional-client relationship	14 (9)
	Societal expectations	13 (8)
Belonging	The Group	52 (33)
	Group Collective Identity	15 (9)
	The profession in relation to other professions	7 (4)
	“Thinking of oneself as a….”	9 (6)
	Doing, being, becoming, belonging to a discipline	3 (2)
	Organisational identity	12 (8)
	Boundary Crossing	58 (36)
	Boundary Closure	17 (11)
Me	Self	95 (59)
	The stories I tell about myself	9 (6)
	Gender	18 (11)
	Sexuality	1 (1)
	Race/Culture	5 (3)
	SES	2 (1)
	Age	3 (2)
	Self in relation to others	5 (3)
	Self in relation to the profession	1 (1)
	Self & Fit	54 (34)
Learning and qualifications	Acquiring knowledge and skills	39 (24)
	Enhancing professional capital	16 (10)
	Shaping or controlling the profession	10 (6)

**Fig. 2:**
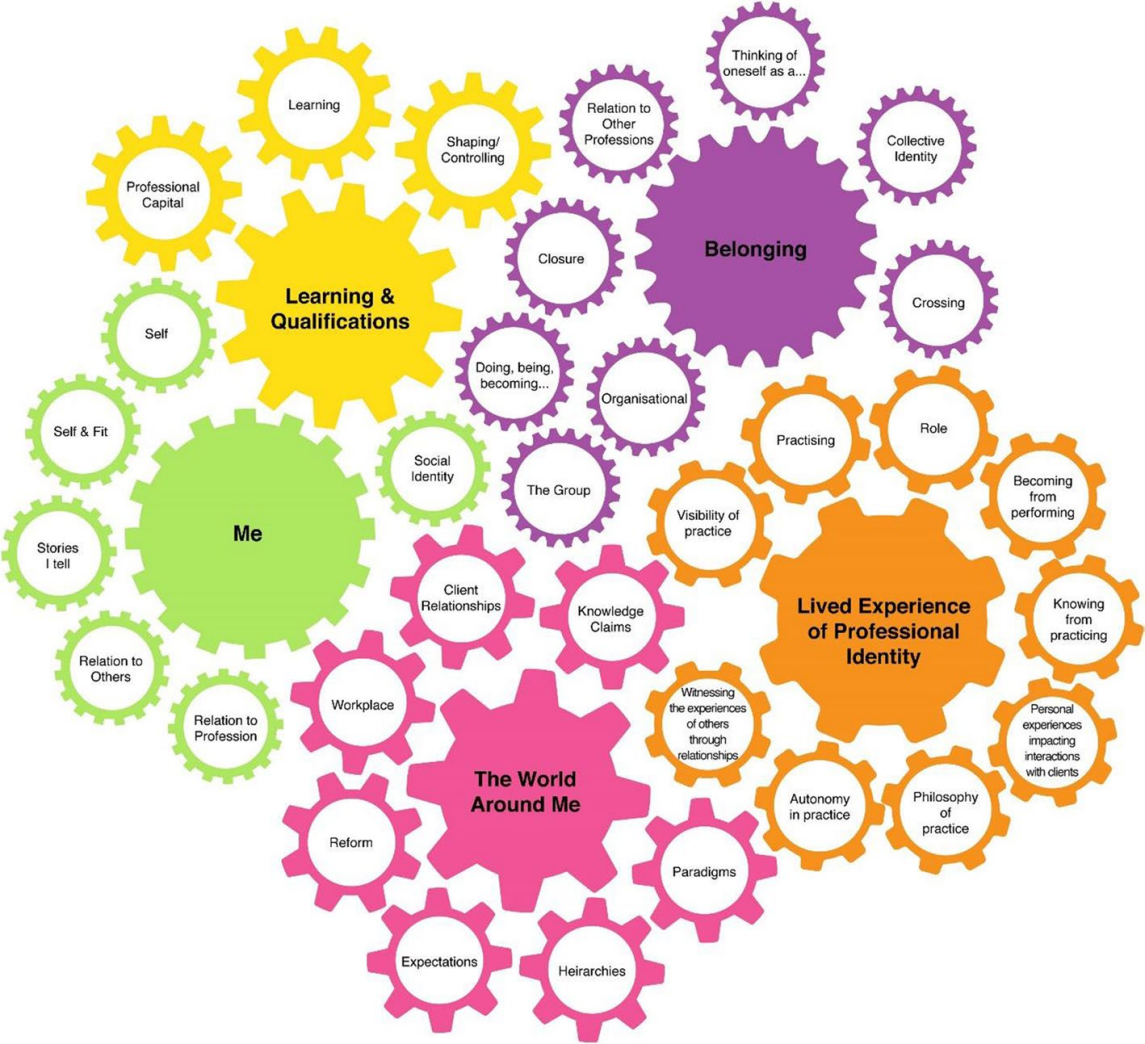
The complex and interrelated nature of constructs of professional identity in the health professions literature

As previously discussed, constructs of professional identity were not discrete but rather intertwined, reciprocal and changing dependent on the individual and circumstances (Fig. 2).

#### The lived experience of professional identity

*The Lived Experience of Professional Identity* comprised three categories, *Becoming from Performing, Knowing from Practising* and *Practising*. *Becoming from Performing was* referred to in 83 or 52% of studies. This category reflects performative aspects of professional identity development with individuals described as learning to identify as health professionals through observation and role modelling which was consolidated through repetition, practice, feedback and validation, and a growing sense of confidence as a health professional. This was not always experienced in a positive way as “the inhibiting culture of nursing was perpetuated through socialisation processes” (Ogilvie, 2012) or lack of role models for novel roles impacted developing identity. *Becoming from performing* also contributed to the co-construction of professional identity through collaboration with other professionals within intra-professional and inter-professional communities of practice.

*Knowing from practising,* discussed in 56 studies (35%), describes health professionals’ experience with patients, clients, communities and students over years of practice giving meaning to and shaping professional identities.

Knowing from practising included two subcategories—*Witnessing the experiences of others through relationships* (30) and *Personal experiences impacting interactions with clients* (7).

*Practising* was described as intrinsic to professional identity in 23 or 14% of studies and was identified as a locus of professional identity in the health professions. Clinical practice was also described as important in the development of leadership and management identity, giving meaning to leadership and a contributing to maintenance of professional collective identity. Three subcategories of *Practicing* were identified in the literature as impacting professional identity development—*Philosophy of practice, Visibility of practice* and *Autonomy in practice.*

*Role* was identified as an important component of professional identity across a number of themes and was referenced specifically in 86 or 54% of studies.

#### The world around me

*Workplace* was described as contributing to professional identity in 62 or 39% or studies. Workplace is described as influencing professional identity by dictating practice of health professionals, through perceived inadequacy of workplace conditions (resources, time, remuneration) and through changing role, changing work, changing work environment and changing practice. Workplace influences on professional identity described above capture influences of political, social and healthcare reforms and advances, however these influences were discussed specifically in 21 or 13% of studies.

*Professional hierarchies* between professions and within professions was described as impacting professional identity in 44 studies. Between-profession power hierarchies described the medical profession at the top of the hierarchy with community midwifery and school nursing literature describing themselves as being low in the hierarchy of professions. Other hierarchies of health professions/specialties were described by academics and by complementary and alternative medicine practitioners who described themselves as professions on the periphery. Within-profession hierarchies were described in the literature in relation to seniority, further training, expanded practice, higher or different qualifications, type of work, place of work (e.g., private vs public), prototypical behaviour in relation to the profession and married vs unmarried female doctors. These dynamics of hierarchy between and within professions were also noted as being important with respect to validation of value and competence of professionals. Hierarchies were noted to exist within an organisational context.

D*ominant paradigms and discourses* of health and practice with its impact on professional identity described in 44 studies. The biomedical model of healthcare was the paradigm most often cited (in 28 of 44 studies) as being the dominant in models of care influencing development of professional identity.

K*nowledge claims* was described as influencing professional identity development in 22 studies. This included the privileging of evidence-based knowledge over experience-based knowledge, and benchmarked, marketable and externally levied ‘quality’ criteria being valued over immeasurable dimensions of practice such as the relational and experiential aspects of healthcare.

The *health professional-client relationship* was also discussed in 14 studies as a component of professional identity underpinning identities such ‘expert’ and ‘fixer’.

S*ocietal expectations* of the health professions*,* were also identified as influencing professional identity formation in 13 or 8% of studies.

#### Belonging

Group identification (*The Group*) as an important aspect of professional identity was identified in 52 or 33% of studies with group collective identity being seen as important in 15 studies. *The profession in relation to other professions,* was another aspect of collective professional identity in seven studies. “Thinking of oneself as a …..” and doing, being, becoming, belonging to a discipline were identified as important in nine and three studies respectively. Identification with the organisation (*Organisational identity*) was seen as an important aspect in professional identity in 12 studies.

Boundaries in the professions were identified as influencing professional identity in 75 papers and described in two ways—through B*oundary Crossing* and through B*oundary Closure*. *Boundary Crossing* was documented in 58 or 36% of studies impacting professional identity in relation to increasing experience (crossing from novice to expert), undertaking more training or qualifications, as well expanded or specialist practice. Transition from clinician roles were also documented as influences—from clinician to educator, from clinician to academic and from clinician to manager/leader. Changes in clinical professions or working in novel areas of practice were also identified as boundary crossing as were dual roles such as clinician-scientists and clinician-manager and working across multiple boundaries, all of which were identified as influencing professional identity. The creation or existence of *boundary closure* between professions or specialties to consolidate professional identity development was discussed in 17 studies.

#### Me

*Self* as a component of professional identity was discussed in 38 (24%) studies. *Self* reflected the foundations of personal identity such as personal characteristics, values, feelings, as well as personal life with strong interrelationships between self, personal and professional identities described. Social work and nursing describe their professions as being intrinsic to self. Another aspect of self relating to professional identity described in nine studies *The stories I tell about myself* and described/included the dominant stories professionals tell about themselves on the basis of their lived experience in the world. These stories, through a reflexive practice, were described as providing opportunities for identification and deconstruction of discourses at play in the narratives with which we construct our professional identity, facilitating professional growth and transformation.

*Gender*, R*ace and Culture, Age,* and *Socioeconomic Status* were described in 18, 5, 2 and 1 study respectively in relation to professional identity. *Self in relation to others* was identified as a category within this theme in five studies with narratives of oneself being described as always being in relation to, and constituted by, ‘other’—supervisors, colleagues, students and family. *Relation to others* was also described in conceptualisations of professional identity related to the caring aspects of nursing/nursing education interactions—including representing, advocating for and providing for others.

Tensions exist between the self and developing professional identity and professional identity construction is partially triggered by work-identity integrity violations. *Self & Fit* was described in a total of 54 or 34% of studies as being important in professional identity. For example, *Self & Fit* was described as impacting professional identity in relation to fit, or not, between self and work, role or practice expectations, as related to fit with members of the professional group as well as fit with the profession.

#### Learning and qualifications

Learning through the *acquiring knowledge and skills* was discussed in 39 or 24% of studies. Further learning and qualifications was described as important in the differentiation of self from others within the profession with lack of further training and learning opportunities described as hampering professional identity development. *Qualifications or credentialing as enhancing professional capital* or improving the status of the health professional was discussed in 16 or 10% of papers. *Qualifications or credentialling as shaping or controlling the profession* were discussed as an influence on professional identity in 10 or 6% or studies.

## Discussion

This scoping review sought to explore and interrogate the literature on professional identity for practising health professionals in order to understand on what disciplines and career stage the evidence is focussed, why the research was undertaken, which theory or framework was used to guide the research and what constructs are used to discuss professional identity. The findings provide insight into professional identity across 17 health professions of 32 investigated. Overall, the majority of studies were in nursing and medicine early in careers with the allied health professions poorly represented. Novel to this review, we identified the role of learning and qualifications as contributing to professional identity through increased knowledge and skills and social capital of the profession. Despite the number of studies and the demonstration of commonalities across the professions, this review demonstrates gaps in the research, both in the number of health professions that are represented in the literature as well as issues with theoretical perspectives and critical aspects of the research. Highlighted as well is the imperative for researcher reflexivity including an interrogation of how the agenda for the research into professional identity is set. Future research must further refine theoretical frameworks to develop questions and guide data collection and its analysis (Merriam & Tisdell, 2015). While there is promising new literature emerging (Devery et al., 2018; Hammond et al., 2016; Scanlan & Hazelton, 2019; Jiang et al., 2019) there is a need for further research that explores professional identity in post-registration allied health professionals. However, these findings provide guidance for health workforces, as individuals and collective identities, to successfully negotiate the constantly changing face of healthcare.

Our review scoped the rationale for research into professional identity, addressing the question of ‘why’ in professional identity research. This synthesis has developed a deeper understanding of the broader contexts and underlying perspectives, assumptions and biases of the research and the researchers. This not only contributes to a more nuanced and critical understanding of the limitations of the literature but also contributes to an understanding of conceptualisations of professional identity across the health professions. Interrogating the rationale for the research also calls to attention to the range of issues that are significant to the health professions and their professional identity, for example the impact of role transition or organisational change on professional identity or links between knowledge, credentials and professional identity. This scoping review has identified that professional identity research in the health professions is largely conducted to explore the impact of political, social and healthcare reforms and advances and to support the development of professional identity. This understanding can guide future research, professional development and education that is pertinent to the health professions including recruitment and retention of workforce, teamwork, progression to academia and leadership.

Highlighting the theoretical framing of professional identity research in this review further contributes to the understanding of the way in which professional identity is understood and discussed in the health professions literature. Our review identified that only 35% of research into professional identity explicitly states a framework or theory of identity. This may reflect a lack of coherence around important theoretical considerations underpinning much of the research and should caution the reader in interpretation of the research. Drilling down further into discussions of professional identity five categories of constructs of professional identity were identified across a broad range of health professions. As well as reflecting the broad categories of individual, social, narrative and poststructural and critical theories of identity, these multiple categories with their multiple subcategories reflect the multi-faceted and nuanced nature of professional identity and provide broad and rich insight into professional identity across the health professions. Taking into account the breadth of these aspects of professional identity will be useful for informing future professional identity research that is relevant to the health professions and further highlights that the arbitrary frameworks must be used with caution with a recognition of the complexity and depth of concepts comprising each category.

Our scoping review reinforces themes identified in previous reviews on professional identity in interprofessional teams and in studies which have included students. Themes of self, the impact of relationships with clients and other health professionals, as well as clinical experience and practice were identified as important influences on the professional identity in this review and others (Best & Williams, 2019; Volpe et al., 2019). As the research into professional identity in the health professions is important it is imbued with power (Zembylas, 2003) and this is reflected in the findings which describe hierarchies, dominant paradigms, contested knowledge, and social expectations. This scoping review also describes the role of hierarchies, autonomy and role enactment in professional identity and affirms existing evidence (Best & Williams, 2019; Volpe et al., 2019). The scoping review also highlights the limited exploration of race and indigeneity, socioeconomic status and gender in professional identity research which has been previously raised as a much needed area of professional identity research in health professions (Sarraf-Yazdi et al., 2021; Tsouroufli et al., 2011; Volpe et al., 2019; Wyatt et al., 2020, 2021a, 2021b).

## Limitations

Due to the small number of studies within most health professions in this scoping review it was not possible to sort theories and constructs of professional identity by profession and thus to identify if aspects of professional identity were more important to some professions than others. In addition, we recognise that categorisation of the theories used may not fully reflect the complex and interrelated nature of aspects of professional identity. Many studies included interwoven aspects to the research, often with one category of identity within another. It was at times difficult to differentiate between aspects of the social such as the group and group-defining behaviours and role with its expectations. For example, “what it means to be and act like a nurse” could be potentially interpreted as a Social Identity Approach (the group) or an Identity Approach (role).

## Conclusion

This scoping review makes an important contribution to the literature by comprehensively examining the rationale and theoretical underpinnings of professional identity research across the health professions as well as exploring the multi-faceted and nuanced nature of professional identity. Professional identity research is under-represented in many health professions and is poorly theorised limiting the cohesion of research across a broad range of health profession. Critical perspectives of professional identity in the health professions literature is lacking, particularly with respects to race and indigeneity, socioeconomic status and gender. Addressing these limitations and taking the broad nature of professional identity into consideration will impact the articulation of meaningful questions and theoretical frameworks for future research.

## Appendix 1 Example search strategy—scopus

**Table:** 

Parent term	Related terms
Professional identity	Professional PRE/3 identit*
AND	
Various (32) health professions (included psychologist; doctor; nurse; midwife; dietitian; physiotherapist; occupational therapist; speech therapist; podiatrist; social worker; pharmacist; optometrist; paramedic; dentist; allied health professional; audiologist; osteopath; chiropractor; Chinese medicine practitioner; naturopath; exercise physiologist; orthotist; prosthetist; orthoptist; perfusion technician; medical radiation practitioner; rehabilitation counsellor; music therapist; art therapist; radiographer; imaging technologist; sonographer; genetic counsellor)	psychologist* OR “psychology graduate*” OR “psychology student*” OR doctor* OR medicine OR “medical student*” OR physician* OR nurs* OR midwif* OR “allied health” OR dieti?ian* OR dietetic* OR physiotherapist* OR “physical therapist*” OR “occupational therap*” OR “speech therap*” OR “speech patholog*” OR podiatr* OR “social work*” OR pharmac* OR paramed* OR dent* OR audiolog* OR osteopath* OR “exercise physiolog*” OR orthot* OR prosthet* OR optometr* OR orthopt* OR “rehabilitation counsel*” OR “music therap*” OR “art therap*” OR radiograph* OR “radiation therap*” OR “imaging technolog*” OR “nuclear medicine” OR “nuclear medicine technolog*” OR ultrasonograph* OR “perfusion techn*” OR “genetic counsel*” OR chiroprac* OR “Chinese medicine pract*” OR naturopath*

## Appendix 2 References included in the scoping review (160)

Allen, B. C. (2011). The role of professional identity commitment in understanding the relationship between casual employment and perceptions of career success. *Career Development International*, 16 (2), 195–216. https://doi.org/10.1108/13620431111115631

Apker, J. & Eggly, S. (2004). Communicating professional identity in medical socialization: considering the ideological discourse of morning report. *Qualitative Health Research*, 14 (3), 411–429. http://search.ebscohost.com/login.aspx?direct=true&db=jlh&AN=106760474&site=ehost-live; https://journals.sagepub.com/doi/abs/10.1177/1049732303260577

Arai, K., Saiki, T., Imafuku, R., Kawakami, C., Fujisaki, K., et al. (2017). What do Japanese residents learn from treating dying patients? The implications for training in end-of-life care. *BMC Medical Education*, 17 (1), 205. https://doi.org/10.1186/s12909-017-1029-6

Baathe, F. N., L. E. (2013). Engaging physicians in organisational improvement work. *Journal of Health, Organisation and Management*, 27 (4), 479–497. https://doi.org/10.1108/JHOM-02-2012-0043

Barbour, J. B. L., J. C. (2015). Measuring professional identity: A review of the literature and a multilevel confirmatory factor analysis of professional identity constructs. *Journal of Professions and Organization*, 2 (1), 38–60. https://doi.org/10.1093/jpo/jou009

Barone, S. M. L.-S., M. (2015). 'Forty bucks is forty bucks': An analysis of a medical Doctor's professional identity. *Language and Communication*, 43, 27–34. https://doi.org/10.1016/j.langcom.2015.04.002

Barraclough, S. J. (2014). Migration of identity of a counsellor educator: using writing as a method of inquiry to explore the in-between spaces. *Reflective Practice*, 15 (3), 363–377. https://doi.org/10.1080/14623943.2014.900013

Bartlett, A. B. (2008). The evolution of the life dream in the development of a psychoanalytic identity: A narrative study of women psychoanalysts. PhD diss Fielding Graduate University Dissertation Abstracts International: Section B: The Sciences and Engineering, 69 (2-B), 1315. http://ovidsp.ovid.com/ovidweb.cgi?T=JS&PAGE=reference&D=psyc6&NEWS=N&AN=2008-99160-509

Becker, B. A. (2013) The Lived Experience of Professional Identity in Master Nursing Academics. University of Minnesota PhD diss https://conservancy.umn.edu/bitstream/handle/11299/155565/1/Becker_umn_0130E_13895.pdf

Beckett, D. G., J. (2004). Perceptions of professional identity: A story from paediatrics. *Studies in Continuing Education*, 26 (2), 195–208. https://doi.org/10.1080/158037042000225218

Beddoe, L. (2013). Health social work: Professional identity and knowledge. *Qualitative Social Work,* 12 (1), 24–40. https://doi.org/10.1177/1473325011415455

Beddoe, L. (2015). Continuing education, registration and professional identity in New Zealand social work. *International Social Work*, 58 (1), 165–174. https://doi.org/10.1177/0020872812473139

Bentley, K. J., Cummings, C. R., Casey, R. C. & Kogut, C. P. (2018). Professional identity and shared decision making among psychiatry residents: designing a brief teaching module. *Journal of Mental Health Training, Education & Practice*, 13 (2), 112–123. https://doi.org/10.1108/JMHTEP-02-2017-0009

Berghout, M. A., Oldenhof, L., Scheer, W. K. & Hilders, C. G. J. M. (2020). From context to contexting: professional identity un/doing in a medical leadership development programme. *Sociology of Health & Illness*, 42 (2), 359–378. https://doi.org/10.1111/1467-9566.13007

Bertin, G. P., M. (2019). Professional identity in community care: The case of specialist physicians in outpatient services in Italy. *Social Science and Medicine*, 226, 21–28. https://doi.org/10.1016/j.socscimed.2019.02.029

Bertrand, S. W. (2009). Inroads to an integrative medicine: case studies of registered nurses' personal use of traditional Chinese medicine. EdD diss University of St Thomas http://search.ebscohost.com/login.aspx?direct=true&db=jlh&AN=109853995&site=ehost-live

Bertrand, S. W. (2010). Inroads to Integrative Health Care: Registered Nurses' Personal Use of Traditional Chinese Medicine Affects Professional Identity and Nursing Practice. *Complementary Health Practice Review*, 15 (1), 14–30. https://doi.org/10.1177/1533210110374639

Birks, M., Chapman, Y. & Francis, K. (2010). Becoming professional by degrees—a grounded theory study of nurses in Malaysian Borneo. *Singapore Nursing Journal*, 37 (3), 31–40. http://search.ebscohost.com/login.aspx?direct=true&db=jlh&AN=105096131&site=ehost-live

Blomberg, H. (2016). Nurses’ blogs as part of a political process – Professional identity as a rhetorical resource for negotiating responsibility and blame. *Discourse, Context and Media*, 13, 82–88. https://doi.org/10.1016/j.dcm.2016.07.001

Blouin, D. (2018). Impact of interpersonal relations on learning and development of professional identity: A study of residents' perceptions. *Emergency Medicine Australasia*, 30 (3), 398–405. https://doi.org/10.1111/1742-6723.12944

Bludau, H. (2017). Hindered Care: Institutional obstructions to carework and professionalism in Czech nursing. *Anthropology of Work Review*, 38 (1), 8–17. https://doi.org/10.1111/awr.12108

Bochatay, N. (2018). Individual and collective strategies in nurses’ struggle for professional identity. *Health Sociology Review*, 27 (3), 263–278. https://doi.org/10.1080/14461242.2018.1469096

Branch, W. T. F., R. (2016). Not all stories of professional identity formation are equal: An analysis of formation narratives of highly humanistic physicians. *Patient Education and Counseling*, 99 (8), 1394–1399. https://doi.org/10.1016/j.pec.2016.03.018

Brenner, P. S., Stets, J. E. & Serpe, R. T. (2021). Introduction: An overview of identities in action. *Identities in Action*, 1–8.

Brosnan, C. & Cribb, A. (2019). Professional identity and epistemic stress: complementary medicine in the academy. *Health Sociology Review*, 28 (3), 307–322. https://doi.org/10.1080/14461242.2019.1678397

Brunton, M. (2017). Risking the Sustainability of the Public Health System: Ethical Conundrums and Ideologically Embedded Reform. *Journal of Business Ethics*, 142 (4), 719–734. https://doi.org/10.1007/s10551-016-3041-x

Carpenter, M. C. P., Sheila (1997). Professional identity for clinical social workers: Impact of changes in health care delivery systems. *Clinical Social Work Journal*, 25 (3), 337–350. https://doi.org/10.1023/A:1025790613308

Carra, K. A., Fortune, T., Ennals, P., D'Cruz, K. & Kohn, H. (2017). Supporting scholarly identity and practice: Narratives of occupational therapy academics. *British Journal of Occupational Therapy*, 80 (8), 502–509. https://doi.org/10.1177/0308022617700653

Carrillo, S. L. & Rubel, D. J. (2019). Connecting With Others: Counselor Educator Identity Development in Hybrid Doctoral Programs. *Journal of Counselor Leadership and Advocacy*, 6 (1), 16–29. https://doi.org/10.1080/2326716X.2018.1545612

Cascón-Pereira, R., Chillas, S. & Hallier, J. (2016). Role-meanings as a critical factor in understanding doctor managers' identity work and different role identities. *Social Science & Medicine*, 170, 18–25. https://doi.org/10.1016/j.socscimed.2016.09.043

Cascón-Pereira, R. & Hallier, J. (2012). Getting that certain feeling: The role of emotions in the meaning, construction and enactment of doctor managers' identities. *British Journal of Management*, 23 (1), 130–144. https://doi.org/10.1111/j.1467-8551.2011.00748.x

Chan, M., Pratt, D., Poole, G. & Sidhu, R. (2018). Professional paradox: identity formation in qualified doctors pursuing further training. *Medical Education*, 52 (3), 302–313. https://doi.org/10.1111/medu.13485

Chang, G. C. (2012). The hidden curriculum: Hazing and professional identity. PhD diss Seattle Pacific University. Dissertation Abstracts International Section A: Humanities and Social Sciences, 72 (11-A), 4323. http://ovidsp.ovid.com/ovidweb.cgi?T=JS&PAGE=reference&D=psyc9&NEWS=N&AN=2012-99090-119

Chow, C. J., Byington, C. L., Olson, L. M., Ramirez, K. P. G., Zeng, S., et al. (2018). A conceptual model for understanding academic physicians' performances of identity: Findings from the University of Utah. *Academic Medicine*, 93 (10), 1539–1549. https://doi.org/10.1097/ACM.0000000000002298

Clandinin, D. J. C., M. (2008). Creating pedagogical spaces for developing doctor professional identity. *Medical Education*, 42 (8), 765–770. http://search.ebscohost.com/login.aspx?direct=true&db=jlh&AN=105688475&site=ehost-live; https://onlinelibrary.wiley.com/doi/abs/10.1111/j.1365-2923.2008.03098.x

Cowin, L. S. J., M.; Craven, R. G.; Marsh, H. W. (2008). Causal modeling of self-concept, job satisfaction, and retention of nurses. *International Journal of Nursing Studies*, 45 (10), 1449–1459. http://search.ebscohost.com/login.aspx?direct=true&db=jlh&AN=105965962&site=ehost-live; https://www.sciencedirect.com/science/article/abs/pii/S0020748907002532?via%3Dihub

Croft, C., Currie, G. & Lockett, A. (2015a). Broken 'two-way windows'? An exploration of professional hybrids. *Public Administration*, 93 (2), 380–394. https://doi.org/10.1111/padm.12115

Croft, C., Currie, G. & Lockett, A. (2015b). The impact of emotionally important social identities on the construction of managerial leader identity: A challenge for nurses in the English National Health Service. *Organization Studies*, 36 (1), 113–131. https://doi.org/10.1177/0170840614556915

Currie, G. F., R.; Martin, G. (2010). Role transition and the interaction of relational and social identity: New nursing roles in the English NHS. *Organization Studies*, 31 (7), 941–961. https://doi.org/10.1177/0170840610373199

Curtis, A. D., Andrew (2013). The impact of specialist training on professional identity, organisational membership, organisational commitment, and stress in correctional psychologists. *Journal of Forensic Practice*, 15 (2), 130–140. https://doi.org/10.1108/14636641311322313

Dadich, A. J., Carmen; Robards, Fiona; Bennett, David (2015). How professional identity shapes youth healthcare. *Journal of Health Organization & Management*, 29 (3), 317–342. https://doi.org/10.1108/JHOM-06-2012-0096

Dahl, B. M. C., Anne (2015). Meanings of knowledge and identity in public health nursing in a time of transition: interpretations of public health nurses' narratives. *Scandinavian Journal of Caring Sciences*, 29 (4), 679–687. https://doi.org/10.1111/scs.12196

de Meis, C., de Almedia Souza, C. & da Silva Filho, J. F. (2007). House and street: Narratives of professional identity among nurses. *Journal of Professional Nursing*, 23 (6), 325–328. https://doi.org/10.1016/j.profnurs.2007.06.004

Deppoliti, D. I. (2003) An exploration of how new registered nurses construct their professional identity in hospital settings. PhD diss Syracuse University. https://sigma.nursingrepository.org/bitstream/handle/10755/22428/Dissertation.pdf?sequence=1

Deppoliti, D. (2008). Exploring how new registered nurses construct professional identity in hospital settings. Journal of Continuing Education in Nursing, 39 (6), 255–262. https://doi.org/10.3928/00220124-20080601-03

Devery, H., Scanlan, J. N. & Ross, J. (2018). Factors associated with professional identity, job satisfaction and burnout for occupational therapists working in eating disorders: A mixed methods study. *Australian Occupational Therapy Journal*, 65 (6), 523–532. https://doi.org/10.1111/1440-1630.12503

Diede, T. T. (2018). Professional identity in the lived experience of hospital nurses. PhD diss Washington State University http://ovidsp.ovid.com/ovidweb.cgi?T=JS&PAGE=reference&D=psyc15&NEWS=N&AN=2018-40527-080

Divall, B. (2015). Negotiating competing discourses in narratives of midwifery leadership in the English NHS. *Midwifery*, 31 (11), 1060–1066. https://doi.org/10.1016/j.midw.2015.07.006

Dombeck, M.-T. (2003). Work narratives: gender and race in professional personhood. *Research in Nursing & Health*, 26 (5), 351–65. http://ovidsp.ovid.com/ovidweb.cgi?T=JS&PAGE=reference&D=med5&NEWS=N&AN=14579256

Elvey, R. H., Karen; Hall, Jason (2013). Who do you think you are? Pharmacists' perceptions of their professional identity. *International Journal of Pharmacy Practice*, 21 (5), 322–332. https://doi.org/10.1111/ijpp.12019

Ennals, P., Fortune, T., Williams, A. & D'Cruz, K. (2016). Shifting occupational identity: Doing, being, becoming and belonging in the academy. *Higher Education Research & Development*, 35 (3), 433–446. https://doi.org/10.1080/07294360.2015.1107884

Estrella, K. (2010). Class in context: A narrative inquiry into the impact of social class mobility and identity on class consciousness in the practice of psychotherapy. PhD diss Fielding Graduate University http://ovidsp.ovid.com/ovidweb.cgi?T=JS&PAGE=reference&D=psyc7&NEWS=N&AN=2010-99061-076

Fagermoen, M. S. (1995). The meaning of nurses' work: a descriptive study of values fundamental to professional identity in nursing. PhD diss University of Rhode Island http://search.ebscohost.com/login.aspx?direct=true&db=jlh&AN=109873016&site=ehost-live

Fagermoen, M. S. (1997). Professional identity: values embedded in meaningful nursing practice. *Journal of Advanced Nursing*, 25 (3), 434–441. https://doi.org/10.1046/j.1365-2648.1997.1997025434.x

Feen-Calligan, H. R. (2012). Professional identity perceptions of dual-prepared art therapy graduates. *Art Therapy*, 29 (4), 150–157. https://doi.org/10.1080/07421656.2012.730027

Ferrell, C. D. (2017) Nursing professional identity: The experiences and meanings for nurses in senior leadership positions. PhD diss University of Mississippi Medical Center https://www.proquest.com/openview/1fd2511e82be5f93485d258d0ac8e94f/1.pdf?pq-origsite=gscholar&cbl=18750

Findlow, S. (2012). Higher education change and professional-academic identity in newly 'academic' disciplines: The case of nurse education. *Higher Education*, 63 (1), 117–133. https://doi.org/10.1007/s10734-011-9449-4

Fitzpatrick, N. D. (2004) Customizing professional identity: A model for early career psychologists. PhD diss The University of Texas at Austin. https://www.proquest.com/openview/10d7327378dbf33c45a631e19b53c1b6/1?pq-origsite=gscholar&cbl=18750&diss=y

Fleit, S. A. (2008). The influence of organizational structure on hospital social work practice and professional identity. PhD diss Stony Brook University http://ovidsp.ovid.com/ovidweb.cgi?T=JS&PAGE=reference&D=psyc6&NEWS=N&AN=2009-99090-169

Forenza, B. & Eckert, C. (2018). Social Worker Identity: A Profession in Context. *Social Work*, 63 (1), 17–26. https://doi.org/10.1093/SW/SWX052

Foster, K. & Roberts, C. (2016). The Heroic and the Villainous: a qualitative study characterising the role models that shaped senior doctors' professional identity. *BMC Medical Education*, 16 (1), 206. https://doi.org/10.1186/s12909-016-0731-0

Fragkiadaki, E., Triliva, S., Natsopoulou, O. & Tzanakis, E. (2019). From social workers to socio-therapists: the transformative journey of substance abuse therapists. *Journal of Social Work Practice in the Addictions*. https://doi.org/10.1080/1533256X.2020.1691408

Franco, M. & Tavares, P. (2013). The influence of professional identity on the process of nurses' training: an empirical study. *Leadership in Health Services*, 26 (2), 118–134. https://doi.org/10.1108/17511871311319713

Frechette, J., Bitzas, V., Kilpatrick, K., Aubry, M. & Lavoie-Tremblay, M. (2020). A hermeneutic-phenomenological study of pediatric intensive care unit nurses' professional identity following hospital re-design: lessons learned for managers. *Journal of Nursing Management*. https://doi.org/10.1111/jonm.13012

Furtaw, P. C. (2004) Expertise versus authority: A social-developmental model of early-career clinical psychologists' professional identity and efficacy. PhD diss Rutgers. https://www.proquest.com/openview/edd051ee2545a221625fa70b87648cd8/1?pq-origsite=gscholar&cbl=18750&diss=y

Gent, N. (2017). How are recent changes to primary care mental health provision within the NHS affecting psychodynamic counsellors’ construction and management of their professional identities? A Foucauldian perspective. *Psychodynamic Practice*, 23 (1), 45–57. https://doi.org/10.1080/14753634.2016.1273792

Gomaa, H. R. (1999) The enchanted loom: School psychologists construct their professional roles and identities. PhD diss Michigan State University. https://www.proquest.com/openview/63b16336aa0ca50b3a2646b6a568f104/1?pq-origsite=gscholar&cbl=18750&diss=y

Gregg, M. F. & Magilvy, J. K. (2001). Professional identity of Japanese nurses: bonding into nursing. *Nursing & Health Sciences*, 3 (1), 47–55. http://search.ebscohost.com/login.aspx?direct=true&db=jlh&AN=107066197&site=ehost-live

Hammond, R., Cross, V. & Moore, A. (2016). The construction of professional identity by physiotherapists: a qualitative study. *Physiotherapy*, 102 (1), 71–77. https://doi.org/10.1016/j.physio.2015.04.002

Handy, J., Warren, L., Hunt, M. & Gardner, D. (2020). Managing professional identity within a changing market environment: New Zealand optometrists’ responses to the growth of corporate optometry. *Kotuitui*, 15 (1), 204–216. https://doi.org/10.1080/1177083X.2019.1700137

Hansen, S. E. M., Susan S.; Biery, Nyann; Dostal, Julie (2019). The Emergence of Family Medicine Identity Among First-Year Residents: A Qualitative Study. *Family Medicine*, 51 (5), 413–419. https://doi.org/10.22454/FamMed.2019.450912

Hanson, D. J. A. (2009) The professional identity of occupational therapists: construction, enactment, and valued supports. PhD diss North Dakota State University. https://www.proquest.com/openview/d3e2583c5e6ceb82df527700dd122b67/1?pq-origsite=gscholar&cbl=18750

Harris, A. & Guillemin, M. (2015). Notes on the medical underground: migrant doctors at the margins. *Health Sociology Review*, 24 (2), 163–174. https://doi.org/10.1080/14461242.2014.999403

Hazen, A. C. M., de Groot, E., de Bont, A. A., de Vocht, S., de Gier, J. J., et al. (2018). Learning through boundary crossing: Professional identity formation of pharmacists transitioning to general practice in the Netherlands. *Academic Medicine*, 93 (10), 1531–1538. https://doi.org/10.1097/ACM.0000000000002180

Healey, A. C. (2010). Female perspectives of professional identity and success in the counseling field. PhD diss Old Dominion University. https://digitalcommons.odu.edu/cgi/viewcontent.cgi?article=1059&context=chs_etds

Hedenskog, C., Nilsson, U. & Jaensson, M. (2017). Swedish-Registered Nurse Anesthetists' Evaluation of Their Professional Self. *Journal of Perianesthesia Nursing*, 32 (2), 106–111. https://doi.org/10.1016/j.jopan.2015.07.002

Hendrikx, W. P. M. A. (2018). Priced not praised: Professional identity of GPs within market-oriented healthcare reform. *Journal of Professions and Organization*, 5 (3), 12–27. https://doi.org/10.1093/jpo/jox011

Hercelinskyj, G. C., Mary; Brown, Peter; Phillips, Brian (2014). Perceptions from the front line: Professional identity in mental health nursing. *International Journal of Mental Health Nursing*, 23 (1), 24–32. https://doi.org/10.1111/inm.12001

Hinojosa, T. J. (2012). Identities of Mexican American women in counselor education and supervision doctoral programs. PhD diss The Pennsylvania State University http://ovidsp.ovid.com/ovidweb.cgi?T=JS&PAGE=reference&D=psyc9&NEWS=N&AN=2012-99230-413

Hinojosa, T. J. & Carney, J. V. (2016). Mexican American Women Pursuing Counselor Education Doctorates: A Narrative Inquiry. *Counselor Education and Supervision*, 55 (3), 198–215. https://doi.org/10.1002/ceas.12045

Hurley, J. (2009). A qualitative study of mental health nurse identities: many roles, one profession. *International Journal of Mental Health Nursing*, 18 (6), 383–390. https://doi.org/10.1111/j.1447-0349.2009.00625.x

Hurley, J. & Lakeman, R. (2011). Becoming a psychiatric/mental health nurse in the UK: A qualitative study exploring processes of identity formation. *Issues in Mental Health Nursing*, 32 (12), 745–751. https://doi.org/10.3109/01612840.2011.609634

Iglesias, M. E. L. & De Bengoa Vallejo, R. B. (2011). Personal and interpersonal value system, self-perception and identity of Spanish nurses: A cross-sectional study. *Journal of Beliefs and Values*, 32 (3), 281–294. https://doi.org/10.1080/13617672.2011.627675

Iwasaki, R., Kageyama, M. & Nagata, S. (2018). The structure of the perceived professional identity of Japanese public health nurses. *Public Health Nursing*, 35 (3), 220–227. https://doi.org/10.1111/phn.12395

Jiang, H., Wang, Y., Chui, E. & Xu, Y. (2019). Professional identity and turnover intentions of social workers in Beijing, China: The roles of job satisfaction and agency type. *International Social Work*, 62 (1), 146–160. https://doi.org/10.1177/0020872817712564

Ka-Hi, M., Kippist, L., Sloan, T. & Eljiz, K. (2019). What is the professional identity of allied health managers? *Asia Pacific Journal of Health Management*, 14 (1), 58–67. https://doi.org/10.24083/apjhm.v14i1.219

Kantek, F. Ş., Belkıs (2017). Factors relating to professional self-concept among nurse managers. *Journal of Clinical Nursing*, 26 (23–24), 4293–4299. https://doi.org/10.1111/jocn.13755

Karanikola, M. D., K.; Koutrouba, A.; Giannakopoulou, M.; Papathanassoglou, E. D. E. (2018). A phenomenological investigation of the interplay among professional worth appraisal, self-esteem and self-perception in nurses: The revelation of an internal and external criteria system. *Frontiers in Psychology*, 9 (OCT). https://doi.org/10.3389/fpsyg.2018.01805

Karpetis, G. (2014). Advocating the Clinical Social Work Professional Identity: A Biographical Study. *Journal of Social Work Practice*, 28 (1), 23–41. https://doi.org/10.1080/02650533.2013.806888

Kluijtmans, M., de Haan, E., Akkerman, S. & van Tartwijk, J. (2017). Professional identity in clinician-scientists: brokers between care and science. *Medical Education*, 51 (6), 645–655. https://doi.org/10.1111/medu.13241

Koskiniemi, A., Vakkala, H. & Pietilainen, V. (2019). Leader identity development in healthcare: an existential-phenomenological study. *Leadership in Health Services*, 32 (1), 83–97. https://doi.org/10.1108/LHS-06-2017-0039

Kumpusalo, E. N., L.; Mattila, K.; Virjo, I.; Isokoski, M.; Kujala, S.; Luhtala, R.; Jääskeläinen, M. (1994). Professional Identities of Young Physicians: A Finnish National Survey. *Medical Anthropology Quarterly*, 8 (1), 69–77. https://doi.org/10.1525/maq.1994.8.1.02a00050

Kunhunny, S. & Salmon, D. (2017). The evolving professional identity of the clinical research nurse: A qualitative exploration. *Journal of Clinical Nursing*, 26 (23–24), 5121–5132. https://doi.org/10.1111/jocn.14055

Kyratsis, Y. A., R.; Phillips, N.; Tracey, P.; George, G. (2017). Health systems in transition: Professional identity work in the context of shifting institutional logics. *Academy of Management Journal*, 60 (2), 610–641. https://doi.org/10.5465/amj.2013.0684

Lafleur, L. B. (2007) Counselors' perceptions of identity and attitudinal differences between counselors and other mental health professionals. PhD diss University of New Orleans. https://www.proquest.com/openview/1a8b1ce7e70fd84c1a99fb7713eef660/1?pq-origsite=gscholar&cbl=18750

Landis, T. T. S., B. M.; Shaw, M. R.; Holliday, C. E. (2020). Professional identity and hospital-based registered nurses: A phenomenological study. *Nursing Forum*, vol. 55, no. 3, pp. 389–394. https://doi.org/10.1111/nuf.12440

Larsson, M., Aldegarmann, U. & Aarts, C. (2009). Professional role and identity in a changing society: three paradoxes in Swedish midwives' experiences. *Midwifery*, 25 (4), 373–381. https://doi.org/10.1016/j.midw.2007.07.009

Leigh, J. T. (2014). The process of professionalisation: Exploring the identities of child protection social workers. *Journal of Social Work*, 14 (6), 625–644. https://doi.org/10.1177/1468017313504380

Lévesque, M. N., Lilian; Gaucher, Charles; Molgat, Marc (2019). Social Representation of Social Work in the Canadian Healthcare Setting: Negotiating a Professional Identity. *British Journal of Social Work*, 49 (8), 2245–2265. https://doi.org/10.1093/bjsw/bcz005

Lewis, I. (2004). Gender and professional identity: A qualitative study of social workers practising as counsellors and psychotherapists. *International Journal of Phytoremediation*, 21 (1), 394–407. https://doi.org/10.1111/j.0312-407X.2004.00169.x

Lotan, D. W. (2019). Female nurses: Professional identity in question how female nurses perceive their professional identity through their relationships with physicians. *Cogent Medicine*, 6 (1), 1,666,626. https://doi.org/10.1080/2331205X.2019.1666626

MacIntosh, J. (2002). Reworking professional identity: Processes and perceptions of experienced nurses. PhD diss Dalhouse University. https://www.collectionscanada.gc.ca/obj/s4/f2/dsk4/etd/NQ82576.PDF?is_thesis=1&oclc_number=56875874http://search.ebscohost.com/login.aspx?direct=true&db=jlh&AN=109842798&site=ehost-live

MacIntosh, J. (2003). Reworking professional nursing identity. *Western Journal of Nursing Research*, 25 (6), 725–741. https://doi.org/10.1177/0193945903252419

Mackay, T. & Zufferey, C. (2015). 'A who doing a what?': Identity, practice and social work education. *Journal of Social Work*, 15 (6), 644–661. https://doi.org/10.1177/1468017314549537

Mallon, A. H. (2018) How are we able to be here? A creative and narrative inquiry into ANZATA-registered art therapy practitioner personal histories. PhD diss Western Sydney University. https://researchdirect.westernsydney.edu.au/islandora/object/uws:34804/datastream/PDF/view

Martin, G., Bushfield, S., Siebert, S. & Howieson, B. (2020). Changing Logics in Healthcare and Their Effects on the Identity Motives and Identity Work of Doctors. *Organization Studies*. https://doi.org/10.1177/0170840619895871

Matsui, T., Sato, M., Kato, Y. & Nishigori, H. (2019). Professional identity formation of female doctors in Japan—gap between the married and unmarried. *BMC Medical Education*, 19 (1), 55. https://doi.org/10.1186/s12909-019-1479-0

McCrae, N. A.-J., S.; Laker, C. (2014). Merely a stepping stone? Professional identity and career prospects following postgraduate mental health nurse training. *Journal of Psychiatric & Mental Health Nursing*, 21 (9), 767–773. https://doi.org/10.1111/jpm.12131

McKenzie, R. W., Michelle (2016). The league of extraordinary generalists: a qualitative study of professional identity and perceptions of role of GPs working on a national after-hours helpline in Australia. *BMC Health Services Research*, 16, 1–8. https://doi.org/10.1186/s12913-016-1387-5

McMurray, R. & Pullen, A. (2008). Boundary management, interplexity, and nostalgia: Managing marginal identities in public health working. *International Journal of Public Administration*, 31 (9), 1058–1078. https://doi.org/10.1080/01900690801924231

McNamara, M. S. (2010). Where is nursing in academic nursing? Disciplinary discourses, identities and clinical practice: A critical perspective from Ireland. *Journal of Clinical Nursing*, 19 (5–6), 766–774. https://doi.org/10.1111/j.1365-2702.2009.03079.x

Mellin, E., Hunt, B. & Nichols, L. (2011). Counselor professional identity: Findings and implications for counseling and interprofessional collaboration. *Journal of Counseling and Development*, 89 (2), 140–147. https://doi.org/10.1002/j.1556-6678.2011.tb00071.x

Meyer, E. M. Z., S.; Brienza, R. S. (2015). The development of professional identity and the formation of teams in the Veterans Affairs Connecticut Healthcare System's Center of Excellence in Primary Care Education Program (CoEPCE). *Academic Medicine*, 90 (6), 802–809. https://doi.org/10.1097/ACM.0000000000000594

Mishra, A. N. A., Catherine; Angst, Corey M.; Agarval, Ritu (2012). Electronic health records assimilation and physician identity evolution: An identity theory perspective. *Information Systems Research*, 23 (3), 738–760. https://doi.org/10.1287/isre.1110.0407

Moorhead, B. (2019). Sustaining professional identity during the initial post-qualification period: Implications for retention strategies. *International Social Work*. https://doi.org/10.1177/0020872819836703

Moorhead, B. B., Karen; Bowles, Wendy (2016). Exploring the development of professional identity with newly qualified social workers. *Australian Social Work*, 69 (4), 456–467. https://doi.org/10.1080/0312407X.2016.1152588

Morriss, L. (2014) Accomplishing social work identity in interprofessional mental health teams following the implementation of the Mental Health Act 2007. PhD diss University of Salford (United Kingdom). https://www.proquest.com/openview/4c95a494e220a292dd4d1e1dd1b646e2/1?pq-origsite=gscholar&cbl=2026366&diss=y

Motoike, J. (2003) The professional identity development of Asian American women psychologists: Integrating culture. PhD diss Southern Illinois University. https://www.proquest.com/openview/a69d8273c8946566fcb7e61ceba56091/1?pq-origsite=gscholar&cbl=18750&diss=y

Mousazadeh, S., Yektatalab, S., Momennasab, M. & Parvizy, S. (2019). Impediments to the formation of intensive care nurses' professional identify. *Nursing Ethics*, 26 (6), 1873–1885. https://doi.org/10.1177/0969733018786059

Ng, L., Steane, R. & Scollay, N. (2018). Leadership mindset in mental health. *Australasian Psychiatry*, 26 (1), 95–97. https://doi.org/10.1177/1039856217734676

Ngai, S. S.-y. (2007). Analyzing Hong Kong outreach worker identity: Associated discourses and power mechanisms. *International Journal of Adolescence and Youth*, 13 (4), 311–326. https://doi.org/10.1080/02673843.2007.9747982

Nicacio, M. C., dos Santos Heringer, A. L., Santana Schroeter, M. & Lenho de Figueiredo Pereira, A. (2016). Perception of nurse midwives regarding their professional identity: a descriptive study. *Online Brazilian Journal of Nursing*, 15 (2), 205–214. http://search.ebscohost.com/login.aspx?direct=true&db=jlh&AN=116925324&site=ehost-live

Niemi, A. & Paasivaara, L. (2007). Meaning contents of radiographers' professional identity as illustrated in a professional journal – a discourse analytical approach. *Radiography*, 13 (4), 258–264. http://search.ebscohost.com/login.aspx?direct=true&db=jlh&AN=105988048&site=ehost-live

O’Shea, J. & McGrath, S. (2019). Contemporary factors shaping the professional identity of occupational therapy lecturers. *British Journal of Occupational Therapy*, 82 (3), 186–194. https://doi.org/10.1177/0308022618796777

Ocek, Z. A. & Vatansever, K. (2014). Perceptions of Turkish dentists of their professional identity in a market-orientated system. *International Journal of Health Services*, 44 (3), 593–613. https://doi.org/10.2190/HS.44.3.i

Ogilvie, C. (2012) The identity work of leadership in a professionalised context: The case of nursing. PhD diss University of Warwick (United Kingdom). http://wrap.warwick.ac.uk/53812/

Ong, S. Y., Lee, M., Lee, L. S., Lim, I. & Tham, K. Y. (2019). Tensions in integrating clinician and educator role identities: A qualitative study with occupational therapists and physiotherapists. *BMJ Open*, 9 (2), e024821. https://doi.org/10.1136/bmjopen-2018-024821

Owens, R. A. (2018). Transition experiences of new rural nurse practitioners. *Journal for Nurse Practitioners*, 14 (8), 605–612. https://doi.org/10.1016/j.nurpra.2018.05.009

Pape, G. D., F.; Horvath, Z. (2018). Assessing the professional identity of dental school faculty: An exploratory study. *Journal of Dental Education*, 82 (11), 1140–1145. https://doi.org/10.21815/JDE.018.117

Peter, E., Simmonds, A. & Liaschenko, J. (2018). Nurse's narratives of moral identity: Making a difference and reciprocal holding. *Nursing Ethics*, 25 (3), 324–334. https://doi.org/10.1177/0969733016648206

Piil, K., Kolbaek, R., Ottmann, G. & Rasmussen, B. (2012). The impact of [corrected] expanded nursing practice on professional identify in Denmark. *Clinical Nurse Specialist*, 26 (6), 329–35. https://doi.org/10.1097/NUR.0b013e31826e3f43

Porter, J. & Wilton, A. (2019). Professional Identity of Allied Health Staff. *Journal of Allied Health*, 48 (1), 11–17. http://search.ebscohost.com/login.aspx?direct=true&db=jlh&AN=135424953&site=ehost-live; https://www.ingentaconnect.com/content/asahp/jah/2019/00000048/00000001/art00004;jsessionid=296c7go8q07ox.x-ic-live-03

Pottie, K., Haydt, S., Farrell, B., Kennie, N., Sellors, C., et al. (2009). Pharmacist's identity development within multidisciplinary primary health care teams in Ontario; qualitative results from the IMPACT (+) project. *Research in Social and Administrative Pharmacy*, 5 (4), 319–326. https://doi.org/10.1016/j.sapharm.2008.12.002

Pratt, M. G., Rockmann, K. W. & Kaufmann, J. B. (2006). Constructing professional identity: The role of work and identity learning cycles in the customization of identity among medical residents. *Academy of Management Journal*, 49 (2), 235–262. https://doi.org/10.5465/AMJ.2006.20786060

Real, K., Bramson, R. & Poole, M. S. (2009). The symbolic and material nature of physician identity: Implications for physician–patient communication. *Health Communicatio*n, 24 (7), 575–587. https://doi.org/10.1080/10410230903242184

Reyes Villagomeza, L. (2019) Shifting Paradigms: The Development of Nursing Identity in Foreign-Educated Physicians Retrained as Nurses Practicing in the United States. PhD diss University of South Florida. https://www.proquest.com/openview/e76d9f626a05b66fa8d2146ad9e5f3a1/1?pq-origsite=gscholar&cbl=18750

Sabanciogullari, S. & Dogan, S. (2017). Professional Self-Concept in Nurses and Related Factors: A Sample from Turkey. *International Journal of Caring Sciences*, 10 (3), 1676–1684. http://search.ebscohost.com/login.aspx?direct=true&db=jlh&AN=127731962&site=ehost-live

Salim, A. M. E., B. (2016). Exploring self-perception of community pharmacists of their professional identity, capabilities, and role expansion. *Journal of Research in Pharmacy Practice*, 5 (2), 116–120. https://doi.org/10.4103/2279-042X.179574

Salvatore, D. N., D.; Fattore, G. (2018). Physicians' professional autonomy and their organizational identification with their hospital. *BMC Health Services Research*, 18 (1). https://doi.org/10.1186/s12913-018-3582-z

Sanders, E. A. (2019) A qualitative study of school psychologists' perception and interpretation of their professional identity. PhD diss University Of Nevada, Las Vegas. https://www.proquest.com/openview/55f193b7114b5a744e03e6007ff06754/1?pq-origsite=gscholar&cbl=18750&diss=y

Sawatsky, A. P., Nordhues, H. C., Merry, S. P., Bashir, M. U. & Hafferty, F. W. (2018). Transformative Learning and Professional Identity Formation During International Health Electives: A Qualitative Study Using Grounded Theory. *Academic Medicine*, 93 (9), 1381–1390. https://doi.org/10.1097/ACM.0000000000002230

Sawatsky, A. P., Santivasi, W. L., Nordhues, H. C., Vaa, B. E., Ratelle, J. T., et al. (2020). Autonomy and professional identity formation in residency training: A qualitative study. *Medical Education*. https://doi.org/10.1111/medu.14073

Schubert, S., Rhodes, P. & Buus, N. (2020). Transformation of professional identity: an exploration of psychologists and psychiatrists implementing Open Dialogue. *Journal of Family Therapy*. https://doi.org/10.1111/1467-6427.12289

Sercu, C., Ayala, R. A. & Bracke, P. (2015). How does stigma influence mental health nursing identities? An ethnographic study of the meaning of stigma for nursing role identities in two Belgian Psychiatric Hospitals. *International Journal of Nursing Studies*, 52 (1), 307–316. https://doi.org/10.1016/j.ijnurstu.2014.07.017

Sims, D. (2011). Reconstructing professional identity for professional and interprofessional practice: A mixed methods study of joint training programmes in learning disability nursing and social work. *Journal of Interprofessional Care*, 25 (4), 265–271. https://doi.org/10.3109/13561820.2011.571352

Smith, C. & Boyd, P. (2012). Becoming an academic: The reconstruction of identity by recently appointed lecturers in nursing, midwifery and the allied health professions. *Innovations in Education and Teaching International*, 49 (1), 63–72. https://doi.org/10.1080/14703297.2012.647784

Snelgrove, S. R. (2009). Nursing work in NHS Direct: constructing a nursing identity in the call-centre environment. *Nursing Inquiry*, 16 (4), 355–365. https://doi.org/10.1111/j.1440-1800.2009.00452.x

Stone, S. E., B.; Holmes, D.; Orgren, R.; Qualters, D. (2002). Identifying oneself as a teacher: the perceptions of preceptors. *Medical Education*, 36 (2), 180–180. http://search.ebscohost.com/login.aspx?direct=true&db=jlh&AN=106095962&site=ehost-live; https://onlinelibrary.wiley.com/doi/abs/10.1046/j.1365-2923.2002.01064.x?sid=nlm%3Apubmed

Swickert, M. L. (1997). Perceptions regarding the professional identity of counselor education doctoral graduates in private practice: A qualitative study. *Counselor Education and Supervision*, 36 (4), 332–340. https://doi.org/10.1002/j.1556-6978.1997.tb00399.x

Tahim, A. (2015). Who are we? A qualitative evaluation of trainees’ perspectives on professional identity in oral and maxillofacial surgery. *Perspectives on Medical Education*, 4 (1), 33–38. https://doi.org/10.1007/s40037-015-0156-1

Takashima, R. & Saeki, K. (2019). Practical Actions Shaped by the Internal Structures of Occupational Therapists' Professional Identities. *Open Journal of Occupational Therapy*, 7 (3), 1–16. https://doi.org/10.15453/2168-6408.1567

Thompson, J., Cook, G. & Duschinsky, R. (2018). “I'm not sure I'm a nurse”: A hermeneutic phenomenological study of nursing home nurses' work identity. *Journal of Clinical Nursing,* 27 (5–6), 1049–1062. https://doi.org/10.1111/jocn.14111

Thompson, L. (2005). Emplaced professional identities: Restructuring and the contested space of town nursing skill. *New Zealand Geographer*, 61 (1), 29–39. https://doi.org/10.1111/j.1745-7939.2005.00003.x

Thomson, O. P. P., Nicola J.; Moore, Ann P. (2014). Osteopaths' professional views, identities and conceptions—A qualitative grounded theory study. *International Journal of Osteopathic Medicine*, 17 (3), 146–159. https://doi.org/10.1016/j.ijosm.2013.12.002

Vincifori, E. M., Monica Molinar (2014). Ethical Code and Professional Identity: A Survey on Italian Midwives. *International Journal of Childbirth*, 4 (1), 55–62. https://doi.org/10.1891/2156-5287.4.1.55

Wiles, F. & Vicary, S. (2019). Picturing social work, puzzles and passion: exploring and developing transnational professional identities. *Social Work Education*, 38 (1), 47–62. https://doi.org/10.1080/02615479.2018.1553236

Woo, H. R. (2013) Instrument construction and initial validation: Professional identity scale in counseling (PISC). PhD diss The University of Iowa. https://citeseerx.ist.psu.edu/viewdoc/download?doi=10.1.1.837.593&rep=rep1&type=pdf

Wright, C. R. (2007) An investigation into the professional identities of occupational therapists in higher education. PhD diss Sheffield Hallam University (United Kingdom). https://www.proquest.com/openview/3ac55d814d09c6f606b854399e951f2a/1?pq-origsite=gscholar&cbl=2026366

Yagil, D. & Medler-Liraz, H. (2015). Clinical expert or service provider? Physicians’ identity work in the context of counterprofessional patient requests. *Qualitative Health Research*, 25 (9), 1199–1211. https://doi.org/10.1177/1049732314557088

Zhang, J., Haycock-Stuart, E., Mander, R. & Hamilton, L. (2015). Navigating the self in maternity care: How Chinese midwives work on their professional identity in hospital setting. *Midwifery*, 31 (3), 388–394. https://doi.org/10.1016/j.midw.2014.11.013

Zufferey, C. (2012). ‘Jack of all trades, master of none?’ Social work identity and homelessness in Australian cities. *Journal of Social Work*, 12 (5), 510–527. https://doi.org/10.1177/1468017310393404

## Appendix 3 Constructs of professional identity linked to references in the health professions literature

**Table:** 

Constructs and themes of professional identity in the health professions literature	References
*The Lived Experience of PI*	
Becoming from performing	Fagermoen (1995), Fagermoen (1997), Swickert (1997), Gomaa (1999), Gregg and Magilvy (2001), MacIntosh (2002), Stone et al. (2002), Deppoliti (2003), MacIntosh (2003), Motoike (2003), Pratt et al. (2006), Lafleur (2007), Bartlett (2008), Clandinin and Cave (2008), Cowin et al. (2008), Deppoliti (2008), McMurray and Pullen (2008), Bertrand (2009), Hanson (2009), Healey (2009), Larsson et al. (2009), Pottie et al. (2009), Real et al. (2009), Allen (2011), Hurley and Lakeman (2011), Iglesias and De Bengoa Vallejo (2011), Cascón-Pereira and Hallier (2012), Chang (2012), Feen-Calligan (2012), Hinojosa (2012), Ogilvie (2012), Smith and Boyd (2012), Baathe and Norbäck (2013), Becker (2013), Beddoe (2013), Franco and Tavares (2013), Woo (2013), Karpetis (2014), Vincifori and Molinar (2014), Barone and Lazzaro-Salazar (2015), Dadich et al. (2015), Harris and Guillemin (2015), Mackay and Zufferey (2015), Sercu et al. (2015), Zhang et al. (2015), Branch and Frankel (2016), Ennals et al. (2016), Foster and Roberts (2016), Hammond et al. (2016), Hinojosa and Carney (2016), Moorhead et al. (2016), Carra et al. (2017), Kluijtmans et al. (2017), Kunhunny and Salmon (2017), Blouin (2018), Bochatay (2018), Chow et al. (2018), Diede (2018), Hazen et al. (2018), Hendrikx (2018), Iwasaki et al. (2018), Mallon (2018), Ng et al. (2018), Owens (2018), Thompson et al. (2018), Brosnan and Cribb (2019), Carrillo and Rubel (2019), Fragkiadaki et al. (2019), Hansen et al. (2019), Jiang et al. (2019), Lotan (2019), Mousazadeh et al. (2019), O’Shea and McGrath (2019), Ong et al. (2019), Porter and Wilton (2019), Reyes Villagomeza (2019), Sanders (2019), Takashima and Saeki (2019), Wiles and Vicary (2019), Frechette et al. (2020), Landis et al. (2020), Sawatsky et al. (2020), Schubert et al. (2020)
Knowing from practicing	Fagermoen (1995), Carpenter and Platt (1997), Gomaa (1999), Gregg and Magilvy (2001), Stone et al. (2002), Beckett and Gough (2004), Furtaw (2004), Pratt et al. (2006), Clandinin and Cave (2008), Cowin et al. (2008), Hanson (2009), Healey (2009), Larsson et al. (2009), Pottie et al. (2009), Real et al. (2009), Snelgrove (2009), Hurley and Lakeman (2011), Feen-Calligan (2012), Becker (2013), Beddoe (2013), Barraclough (2014), Karpetis (2014), Morriss (2014), Thomson et al. (2014), Vincifori and Molinar (2014), Barone and Lazzaro-Salazar (2015), Dadich et al. (2015), Mackay and Zufferey (2015), Sercu et al. (2015), Zhang et al. (2015), Ennals et al. (2016), Hammond et al. (2016), Moorhead et al. (2016), Nicacio et al. (2016), Arai et al. (2017), Hedenskog et al. (2017), Kluijtmans et al. (2017), Kunhunny and Salmon (2017), Kyratsis et al. (2017), Sabanciogullari and Dogan (2017), Chow et al. (2018), Forenza and Eckert (2018), Hendrikx (2018), Iwasaki et al. (2018), Owens (2018), Peter et al. (2018), Sawatsky et al. (2018), Carrillo and Rubel (2019), Fragkiadaki et al. (2019), Hansen et al. (2019), Jiang et al. (2019), Mousazadeh et al. (2019), O’Shea and McGrath (2019), Porter and Wilton (2019), Sanders (2019), Takashima and Saeki (2019), Landis et al. (2020), Schubert et al. (2020)
Witnessing experiences of others through relationships	Carpenter and Platt (1997), Fagermoen (1997), Beckett and Gough (2004), Furtaw (2004), Clandinin and Cave (2008), Cowin et al. (2008), Hurley (2009), Larsson et al. (2009), Hurley and Lakeman (2011), Baathe and Norbäck (2013), Karpetis (2014), Leigh (2014), Vincifori and Molinar (2014), Barone and Lazzaro-Salazar (2015), Dadich et al. (2015), Sercu et al. (2015), Zhang et al. (2015), Branch and Frankel (2016), McKenzie and Williamson (2016), Arai et al. (2017), Ferrell (2017), Kyratsis (2017), Diede (2018), Peter et al. (2018), Sawatsky et al. (2018), Bertin and Pantalone (2019), Hansen et al. (2019), Lévesque et al. (2019), Takashima and Saeki (2019), Schubert et al. (2020)
Personal experiences impacting interactions with clients	Swickert (1997), Gomaa (1999), Motoike (2003), Bertrand (2009), Bertrand (2010), Ferrell (2017), Sawatsky et al. (2018)
Practising	Fagermoen (1995), Gregg and Magilvy (2001), Deppoliti (2003), Cowin et al. (2008), Deppoliti (2008), McNamara (2010), Sims (2011), Cascón-Pereira and Hallier (2012), Ogilvie (2012), Smith and Boyd (2012), Baathe and Norbäck (2013), Franco and Tavares (2013), Croft (2015a, 2015b), Divall (2015), Nicacio et al. (2016), Carra et al. (2017), Ferrell (2017), Kluijtmans et al. (2017), Kunhunny and Salmon (2017), Mallon (2018), Brosnan and Cribb (2019), Koskiniemi et al. (2019), Landis et al. (2020)
Philosophy of practice	Carpenter and Platt (1997), Swickert (1997), Gregg and Magilvy (2001), MacIntosh (2002, 2003), Beckett and Gough (2004), Ngai (2007), Niemi and Paasivaara (2007), Wright (2007), Bartlett (2008), Clandinin and Cave (2008), Hanson (2009), Healey (2009), Hurley (2009), Pottie et al. (2009), Snelgrove (2009), Zufferey (2012), Karpetis (2014), Leigh (2014), McCrae et al. (2014), Ocek and Vatansever (2014), Thomson et al. (2014), Dadich et al. (2015), Dahl and Clancy (2015), Mackay and Zufferey (2015), Sercu et al. (2015), Zhang et al. (2015), Branch and Frankel (2016), Nicacio et al. (2016), Bludau (2017), Ferrell (2017), Kyratsis et al. (2017), Diede (2018), Forenza and Eckert (2018), Sawatsky et al. (2018), Thompson et al. (2018), Lévesque (2019), Takashima and Saeki (2019)
Visibility of practice	Ngai (2007), Hanson (2009), Snelgrove (2009), Hercelinskyj et al. (2014), Karpetis (2014), Morriss (2014), Kunhunny and Salmon (2017), Bochatay (2018), Diede (2018), Mallon (2018), Thompson et al. (2018), Lévesque et al. (2019), Takashima and Saeki (2019), Landis et al. (2020)
Autonomy in practice	Swickert (1997), MacIntosh (2002, 2003), Furtaw (2004), Ngai (2007), Fleit (2008), McMurray and Pullen (2008), Hanson (2009), Larsson et al. (2009), Currie et al. (2010), McNamara (2010), Findlow (2012), Piil et al. (2012), Zufferey (2012), Baathe and Norbäck (2013), Morriss (2014), Ocek and Vatansever (2014), Sercu et al. (2015), Cascón-Pereira et al. (2016), Brunton (2017), Gent (2017), Ng et al. (2018), Owens (2018), Salvatore et al. (2018), O’Shea and McGrath (2019), Frechette et al. (2020), Landis et al. (2020), Martin et al. (2020), Sawatsky et al. (2020)
Role	Kumpusalo et al. (1994), Swickert (1997), Gomaa (1999), MacIntosh (2002, 2003), Motoike (2003), Beckett and Gough (2004), Furtaw (2004), Thompson (2005), Pratt et al. (2006), de Meis et al. (2007), Lafleur (2007), Wright (2007), Fleit (2008), McMurray and Pullen (2008), Hanson (2009), Healey (2009), Hurley (2009), Larsson et al. (2009), Pottie et al. (2009), Real et al. (2009), Snelgrove (2009), Currie et al. (2010), Allen (2011), Hurley and Lakeman (2011), Mellin et al. (2011), Sims (2011), Cascón-Pereira and Hallier (2012), Findlow (2012), Mishra et al. (2012), Ogilvie (2012), Piil et al. (2012), Smith and Boyd (2012), Zufferey (2012), Elvey et al. (2013), Franco and Tavares (2013), Woo (2013), Hercelinskyj et al. (2014), Karpetis (2014), Leigh (2014), Morriss (2014), Thomson et al. (2014), Barbour and Lammers (2015), Barone and Lazzaro-Salazar (2015), Croft (2015a, 2015b), Dahl and Clancy (2015), Mackay and Zufferey (2015), Meyer et al. (2015), Tahim (2015), Yagil and Medler-Liraz (2015), Zhang et al. (2015), Cascón-Pereira et al. (2016), Ennals et al. (2016), Hammond et al. (2016), McKenzie and Williamson (2016), Moorhead et al. (2016), Salim and Elgizoli (2016), Arai et al. (2017), Ferrell (2017), Kantek and Şimşek (2017), Kluijtmans et al. (2017), Kunhunny and Salmon (2017), Kyratsis et al. (2017), Forenza and Eckert (2018), Hazen et al. (2018), Hendrikx (2018), Iwasaki et al. (2018), Mallon (2018), Owens (2018), Pape et al. (2018), Salvatore (2018), Sawatsky et al. (2018), Thompson et al. (2018), Bertin and Pantalone (2019), Fragkiadaki et al. (2019), Hansen et al. (2019), Ka-Hi et al. (2019), Moorhead (2019), Ong et al. (2019), Porter and Wilton (2019), Sanders (2019), Wiles and Vicary (2019), Berghout et al. (2020), Frechette et al. (2020), Handy et al. (2020)
*The World Around Me*	
Workplace and the organisation	Fagermoen (1995), Carpenter and Platt (1997), MacIntosh (2002), Deppoliti (2003), MacIntosh (2003), Motoike (2003), Beckett and Gough (2004), Fitzpatrick (2004), Furtaw (2004), Thompson (2005), Pratt et al. (2006), de Meis et al. (2007), Wright (2007), Bartlett (2008), Deppoliti (2008), Fleit (2008), McMurray and Pullen (2008), Hanson (2009), Larsson et al. (2009), Pottie et al. (2009), Real et al. (2009), Sims (2011), Cascón-Pereira and Hallier (2012), Chang (2012), Feen-Calligan (2012), Findlow (2012), Piil et al. (2012), Smith and Boyd (2012), Zufferey (2012), Baathe and Norbäck (2013), Beddoe (2013), Hercelinskyj et al. (2014), Leigh (2014), Ocek and Vatansever (2014), Beddoe (2015), Dadich et al. (2015), Harris and Guillemin (2015), Sercu et al. (2015), Zhang et al. (2015), Hammond et al. (2016), McKenzie and Williamson (2016), Moorhead et al. (2016), Bludau (2017), Kantek and Şimşek (2017), Kyratsis et al. (2017), Devery et al. (2018), Hazen et al. (2018), Iwasaki et al. (2018), Ng et al. (2018), Owens (2018), Pape (2018), Fragkiadaki et al. (2019), Jiang et al. (2019), Koskiniemi et al. (2019), Moorhead (2019), Mousazadeh et al. (2019), Ong et al. (2019), Porter and Wilton (2019), Frechette et al. (2020), Handy et al. (2020), Landis et al. (2020), Martin et al. (2020)
Political, social and healthcare reforms and advances	Carpenter and Platt (1997), Swickert (1997), Thompson (2005), Hurley and Lakeman (2011), Findlow (2012), Mishra et al. (2012), Ogilvie (2012), Zufferey (2012), Ocek and Vatansever (2014), Blomberg (2016), Bludau (2017), Brunton (2017), Gent (2017), Forenza and Eckert (2018), Hendrikx (2018), Iwasaki et al. (2018), Sawatsky (2018), Fragkiadaki et al. (2019), Wiles and Vicary (2019), Frechette et al. (2020), Martin et al. (2020)
Professional hierarchies	MacIntosh (2002), Deppoliti (2003), MacIntosh (2003), Apker and Eggly (2004), Lewis (2004), Thompson (2005), de Meis et al. (2007), Deppoliti (2008), McMurray and Pullen (2008), Currie et al. (2010), McNamara (2010), Feen-Calligan (2012), Findlow (2012), Ogilvie (2012), Piil et al. (2012), Beddoe (2013), Karpetis (2014), Morriss (2014), Ocek and Vatansever (2014), Thomson et al. (2014), Beddoe (2015), Croft (2015a, 2015b), Divall (2015), Meyer et al. (2015), Sercu et al. (2015), Foster and Roberts (2016), McKenzie (2016), Salim and Elgizoli (2016), Kunhunny and Salmon (2017), Kyratsis et al. (2017), Chan et al. (2018), Diede (2018), Ng et al. (2018), Thompson et al. (2018), Brosnan and Cribb (2019), Ka-Hi et al. (2019), Lotan (2019), Matsui et al. (2019), Mousazadeh et al. (2019), Frechette et al. (2020), Landis et al. (2020), Martin et al. (2020), Schubert et al. (2020) Between-profession power hierarchies described the medical profession at the top of the hierarchy (Deppoliti, 2003; Deppoliti, 2008; Larsson et al., 2009; Beddoe, 2013; Divall, 2015; Blomberg, 2016; Hammond et al., 2016; Lotan, 2019; Mousazadeh et al., 2019; Wiles & Vicary, 2019; Landis et al., 2020)
Dominant paradigms	Swickert (1997), Gomaa (1999), Dombeck (2003), Apker and Eggly (2004), Beckett and Gough (2004), Lewis (2004), de Meis et al. (2007), Ngai (2007), McMurray and Pullen (2008), Hanson (2009), Larsson et al. (2009), Real et al. (2009), Currie et al. (2010), Findlow (2012), Ogilvie (2012), Piil et al. (2012), Beddoe (2013), Morriss (2014), Ocek and Vatansever (2014), Thomson et al. (2014), Barone and Lazzaro-Salazar (2015), Beddoe (2015), Meyer et al. (2015), Sercu et al. (2015), Yagil and Medler-Liraz (2015), Zhang et al. (2015), Blomberg (2016), Arai et al. (2017), Gent (2017), Kyratsis et al. (2017), Bentley et al. (2018), Devery et al. (2018), Mallon (2018), Ng et al. (2018), Peter et al. (2018), Thompson et al. (2018), Brosnan and Cribb (2019), Ka-Hi et al. (2019), Lévesque et al. (2019), Matsui et al. (2019), Wiles and Vicary (2019), Berghout et al. (2020), Landis et al. (2020), Schubert et al. (2020)
Knowledge claims	MacIntosh (2002), Deppoliti (2003), MacIntosh (2003), Ngai (2007), Deppoliti (2008), McMurray and Pullen (2008), Larsson et al. (2009), McNamara (2010), Findlow (2012), Ogilvie (2012), Thomson et al. (2014), Zhang et al. (2015), Nicacio et al. (2016), Gent (2017), Kunhunny and Salmon (2017), Devery et al. (2018), Brosnan and Cribb (2019), Mousazadeh et al. (2019), O’Shea and McGrath (2019), Takashima and Saeki (2019), Landis et al. (2020), Schubert et al. (2020)
Health professional-client relationship	Fagermoen (1997), Ngai (2007), Barone and Lazzaro-Salazar (2015), Yagil and Medler-Liraz (2015), Zhang et al. (2015), Branch and Frankel (2016), Arai et al. (2017), Bentley et al. (2018), Diede (2018), Peter et al. (2018), Lévesque et al. (2019), Takashima and Saeki (2019), Handy et al. (2020), Schubert et al. (2020)
Societal expectations	MacIntosh (2002), Dombeck (2003), MacIntosh (2003), Motoike (2003), Lewis (2004), de Meis et al. (2007), Ngai (2007), Larsson et al. (2009), Healey (2010), Bentley et al. (2018), Lotan (2019), Matsui et al. (2019), Mousazadeh et al. (2019)
*Belonging*	
The Group	Fagermoen (1997), Gomaa (1999), Gregg and Magilvy (2001), Deppoliti (2003), Lafleur (2007), Niemi and Paasivaara (2007), Bartlett (2008), Cowin et al. (2008), Deppoliti (2008), Hanson (2009), Healey (2009), Hurley (2009), Real et al. (2009), McNamara (2010), Iglesias and De Bengoa Vallejo (2011), Mellin et al. (2011), Cascón-Pereira and Hallier (2012), Hinojosa (2012), Mishra et al. (2012), Ogilvie (2012), Zufferey (2012), Elvey et al. (2013), Woo (2013), Karpetis (2014), McCrae et al. (2014), Vincifori and Molinar (2014), Barbour and Lammers (2015), Croft (2015a, 2015b), Hammond (2015), Mackay and Zufferey (2015), Meyer et al. (2015), Cascón-Pereira et al. (2016), Ennals et al. (2016), Hinojosa and Carney (2016), Carra et al. (2017), Ferrell (2017), Kunhunny and Salmon (2017), Blouin (2018), Bochatay (2018), Forenza and Eckert (2018), Hendrikx (2018), Ng et al. (2018), Thompson et al. (2018), Carrillo and Rubel (2019), Jiang et al. (2019), Moorhead (2019), Mousazadeh et al. (2019), O’Shea and McGrath (2019), Porter and Wilton (2019), Takashima and Saeki (2019), Wiles and Vicary (2019)
Group Collective Identity	Gregg and Magilvy (2001), de Meis et al. (2007), Bartlett (2008), Fleit (2008), McNamara (2010), Findlow (2012), Ogilvie (2012), Leigh (2014), McCrae et al. (2014), Mackay and Zufferey (2015), Blouin (2018), Iwasaki et al. (2018), Mousazadeh et al. (2019), Porter and Wilton (2019), Martin et al. (2020)
The profession in relation to other professions	Larsson et al. (2009), Piil et al. (2012), Beddoe (2015), Croft (2015a, 2015b), Salim and Elgizoli (2016), Ferrell (2017)
“Thinking of oneself as a….”	Stone et al. (2002), Beckett and Gough (2004), Birks et al. (2010), Cascón-Pereira and Hallier (2012), Chang (2012), Bentley et al. (2018), Chan et al. (2018), Berghout et al. (2020), Sawatsky et al. (2020)
Doing, being, becoming, belonging to a discipline	Fagermoen (1995), McCrae et al. (2014), Nicacio et al. (2016)
Organisational identity	Curtis and Day (2013), Franco and Tavares (2013), McCrae et al. (2014), Barbour and Lammers (2015), Croft (2015a, 2015b), Kyratsis et al. (2017), Salvatore et al. (2018), Thompson et al. (2018), Bertin and Pantalone (2019), Berghout et al. (2020), Martin et al. (2020)
Boundary Crossing	Stone et al. (2002), Beckett and Gough (2004), Furtaw (2004), Thompson (2005), Ngai (2007), Wright (2007), McMurray and Pullen (2008), Larsson et al. (2009), Pottie et al. (2009), Snelgrove (2009), Birks et al. (2010), Currie et al. (2010), Estrella (2010), McNamara (2010), Sims (2011), Cascón-Pereira and Hallier (2012), Findlow (2012), Hinojosa (2012), Ogilvie (2012), Piil et al. (2012), Smith and Boyd (2012), Baathe and Norbäck (2013), Becker (2013), Curtis and Day (2013), Barraclough (2014), Ocek and Vatansever (2014), Croft (2015a, 2015b), Divall (2015), Meyer et al. (2015), Cascón-Pereira et al. (2016), Ennals et al. (2016), Carra et al. (2017), Ferrell (2017), Gent (2017), Hedenskog et al. (2017), Kantek and Şimşek (2017), Kluijtmans et al. (2017), Kyratsis et al. (2017), Chan et al. (2018), Hazen et al. (2018), Hendrikx (2018), Ng et al. (2018), Owens (2018), Salvatore et al. (2018), Bertin and Pantalone (2019), Brosnan and Cribb (2019), Carrillo and Rubel (2019), Hansen et al. (2019), Ka-Hi et al. (2019), Koskiniemi et al. (2019), O’Shea and McGrath (2019), Ong et al. (2019), Reyes Villagomeza (2019), Berghout et al. (2020), Handy et al. (2020), Martin et al. (2020)
Boundary Closure	Swickert (1997), Furtaw (2004), Lafleur (2007), Fleit (2008), Hanson (2009), Hurley (2009), Snelgrove (2009), McNamara (2010), Mellin et al. (2011), Smith and Boyd (2012), Beddoe (2013), Curtis and Day (2013), Hercelinskyj et al. (2014), Beddoe (2015), Tahim (2015), Thompson (2018), Brosnan and Cribb (2019)
*Me*	
Self	Fagermoen (1995), Carpenter and Platt (1997), Gomaa (1999), Stone et al. (2002), Fitzpatrick (2004), Furtaw (2004), Thompson (2005), Wright (2007), Bartlett (2008), Cowin et al. (2008), Bertrand (2009), Healey (2009), Real et al. (2009), Bertrand (2010), Estrella (2010), Hurley and Lakeman (2011), Iglesias and De Bengoa Vallejo (2011), Feen-Calligan (2012), Leigh (2014), McCrae et al. (2014), Morriss (2014), Vincifori and Molinar (2014), Hammond (2015), Sercu et al. (2015), Branch and Frankel (2016), Ferrell (2017), Hedenskog et al. (2017), Sabanciogullari and Dogan (2017), Iwasaki et al. (2018), Karanikola (2018), Peter et al. (2018), Jiang et al. (2019), Koskiniemi et al. (2019), Matsui et al. (2019), Ong et al. (2019), Takashima and Saeki (2019), Wiles and Vicary (2019), Schubert et al. (2020)
The stories I tell about myself	Ngai (2007), Bartlett (2008), Clandinin and Cave (2008), McMurray and Pullen (2008), Barraclough (2014), Branch and Frankel (2016), Carra et al. (2017), Fragkiadaki et al. (2019), Berghout et al. (2020)
Gender	MacIntosh (2002), Dombeck (2003), MacIntosh (2003), Motoike (2003), Lewis (2004), Thompson (2005), de Meis et al. (2007), Healey (2009), Larsson et al. (2009), Hinojosa (2012), Ogilvie (2012), Divall (2015), Hinojosa and Carney (2016), Nicacio et al. (2016), Hedenskog et al. (2017), Chow et al. (2018), Lotan (2019), Matsui et al. (2019)
Sexuality	Hinojosa (2012)
Race/Culture	Dombeck (2003), Estrella (2010), Iglesias and De Bengoa Vallejo (2011), Hinojosa (2012), Hinojosa and Carney (2016) Age, (Larsson et al., 2009; Iglesias & De Bengoa Vallejo, 2011; Hedenskog et al., 2017)
SES	Lewis (2004), Estrella (2010)
Age	Hedenskog et al. (2017), Iglesias and De Bengoa Vallejo (2011), Larsson et al. (2009)
Self in relation to others	Clandinin and Cave (2008), Becker (2013), Barraclough (2014), Ferrell (2017), Blouin (2018)
Self in relation to the profession	Ogilvie (2012)
Self & Fit	Fagermoen (1995), Carpenter and Platt (1997), Gregg and Magilvy (2001), MacIntosh (2002), Deppoliti (2003), MacIntosh (2003), Fitzpatrick (2004), Pratt et al. (2006), Wright (2007), Bartlett (2008), Deppoliti (2008), Fleit (2008), Hanson (2009), Healey (2009), Sims (2011), Cascón-Pereira and Hallier (2012), Chang (2012), Findlow (2012), Smith and Boyd (2012), Zufferey (2012), Becker (2013), Hercelinskyj et al. (2014), Leigh (2014), Morriss (2014), Barbour and Lammers (2015), Croft (2015a, 2015b), Dadich et al. (2015), Sercu et al. (2015), Zhang et al. (2015), Blomberg (2016), Branch and Frankel (2016), Cascón-Pereira et al. (2016), Bludau (2017), Brunton (2017), Ferrell (2017), Gent (2017), Kunhunny and Salmon (2017), Bentley et al. (2018), Bochatay (2018), Forenza and Eckert (2018), Hendrikx (2018), Iwasaki et al. (2018), Peter et al. (2018), Bertin and Pantalone (2019), Bresnen et al. (2019), Fragkiadaki et al. (2019), Ong et al. (2019), Wiles and Vicary (2019), Berghout et al. (2020), Frechette et al. (2020), Handy et al. (2020), Martin et al. (2020), Schubert et al. (2020) For example Self & fit was described as impacting professional identity in relation to fit, or not, between self and work, role or practice expectations, (Gregg & Magilvy, 2001; Deppoliti, 2003; Fitzpatrick, 2004; Pratt et al., 2006; Ngai, 2007; Niemi & Paasivaara, 2007; Wright, 2007; Deppoliti, 2008; Hanson, 2009; Healey, 2010; Sims, 2011; Zufferey, 2012; Woo, 2013; Morriss, 2014; Sercu et al., 2015; Yagil & Medler-Liraz, 2015; Zhang et al., 2015; Cascón-Pereira et al., 2016; Brunton, 2017; Ferrell, 2017; Gent, 2017; Kunhunny & Salmon, 2017; Kyratsis et al., 2017; Forenza & Eckert, 2018; Peter et al., 2018; Jiang et al., 2019; Wiles & Vicary, 2019; Handy et al., 2020; Landis et al., 2020; Schubert et al., 2020) as related to fit with members of the professional group (Cascón-Pereira et al., 2016) as well as fit with the profession. Wright (2007), Zufferey (2012)
*Learning and Qualifications*	
Acquiring knowledge and skills	Gregg and Magilvy (2001), Stone et al. (2002), Deppoliti (2003), Beckett and Gough (2004), Pratt et al. (2006), de Meis et al. (2007), Niemi and Paasivaara (2007), Deppoliti (2008), Bertrand (2009), Pottie et al. (2009), Snelgrove (2009), Bertrand (2010), Birks et al. (2010), Hurley and Lakeman (2011), Sims (2011), Feen-Calligan (2012), Piil et al. (2012), Smith and Boyd (2012), Becker (2013), Curtis and Day (2013), Franco and Tavares (2013), Karpetis (2014), Tahim (2015), Zhang et al. (2015), Arai et al. (2017), Kluijtmans et al. (2017), Sabanciogullari and Dogan (2017), Blouin (2018), Chan et al. (2018), Devery et al. (2018), Owens (2018), Salvatore et al. (2018), Sawatsky et al. (2018), Carrillo and Rubel (2019), Hansen (2019), Jiang et al. (2019), Moorhead (2019), Takashima and Saeki (2019), Schubert et al. (2020)
Enhancing professional capital	Gregg and Magilvy (2001), de Meis et al. (2007), Larsson et al. (2009), Pottie et al. (2009), Snelgrove (2009), Birks et al. (2010), Hurley and Lakeman (2011), Findlow (2012), Beddoe (2013), Franco and Tavares (2013), Karpetis (2014), Tahim (2015), Chan et al. (2018), Ng et al. (2018), Beddoe et al. (2019), Brosnan and Cribb (2019)
Shaping or controlling the profession	de Meis et al. (2007), Lafleur (2007), Feen-Calligan (2012), Findlow (2012), Beddoe (2013), Barraclough (2014), Karpetis (2014), Beddoe (2015), Mackay and Zufferey (2015), Brosnan and Cribb (2019)
